# Single-cell analysis of AIMP2 splice variants informs on drug sensitivity and prognosis in hematologic cancer

**DOI:** 10.1038/s42003-020-01353-x

**Published:** 2020-10-30

**Authors:** Jayoung Ku, Ryul Kim, Dongchan Kim, Daeyoon Kim, Seulki Song, Keonyong Lee, Namseok Lee, MinA Kim, Sung-Soo Yoon, Nam Hoon Kwon, Sunghoon Kim, Yoosik Kim, Youngil Koh

**Affiliations:** 1 Department of Chemical and Biomolecular Engineering and KAIST Institute for Health Science and Technology (KIHST), Korea Advanced Institute of Science and Technology (KAIST), Daejeon, Republic of Korea; 2 Cancer Research Institute, Seoul National University College of Medicine, Seoul, Republic of Korea; 3 Biomedical Research Institute, Seoul National University Hospital, Seoul, Republic of Korea; 4 Department of Internal Medicine, Seoul National University Hospital, Seoul, Republic of Korea; 5 Medicinal Bioconvergence Research Center, Department of Molecular Medicine and Biopharmaceutical Sciences, Graduate School of Convergence Science and Technology, College of Pharmacy, Seoul National University, Seoul, Republic of Korea

**Keywords:** Haematological cancer, Assay systems, Diagnostic markers, Prognostic markers, Alternative splicing

## Abstract

Aminoacyl-tRNA synthetase-interacting multifunctional protein 2 (AIMP2) is a non-enzymatic component required for the multi-tRNA synthetase complex. While exon 2 skipping alternatively spliced variant of AIMP2 (AIMP2-DX2) compromises AIMP2 activity and is associated with carcinogenesis, its clinical potential awaits further validation. Here, we found that AIMP2-DX2/AIMP2 expression ratio is strongly correlated with major cancer signaling pathways and poor prognosis, particularly in acute myeloid leukemia (AML). Analysis of a clinical patient cohort revealed that AIMP2-DX2 positive AML patients show decreased overall survival and progression-free survival. We also developed targeted RNA-sequencing and single-molecule RNA-FISH tools to quantitatively analyze AIMP2-DX2/AIMP2 ratios at the single-cell level. By subclassifying hematologic cancer cells based on their AIMP2-DX2/AIMP2 ratios, we found that downregulating AIMP2-DX2 sensitizes cells to anticancer drugs only for a subgroup of cells while it has adverse effects on others. Collectively, our study establishes AIMP2-DX2 as a potential biomarker and a therapeutic target for hematologic cancer.

## Abstract (web summary)

Ku, Kim et al develop a method to analyse the ratio of the alternatively spliced variant of AIMP2 to full length AIMP via single-molecule RNA-FISH. They can subclassify hematologic cancer based on AIMP2-DX2/AIMP2 ratio and find that cells with high AIMP2-DX2 ratio can be sensitized to chemotherapy drugs by depleting AIMP2-DX2.

## Introduction

Aminoacyl-tRNA synthetase-interacting multifunctional protein 2 (AIMP2) is a component of a macromolecular protein complex consisting of several different aminoacyl-tRNA synthetases (MSC). It is a nonenzymatic auxiliary component required for the integration and stability of this translational complex^1,2^. In addition, AIMP2 can also act as a potent tumor suppressor^3^. In response to DNA damage, a fraction of AIMP2 dissociates from the MSC, and induces apoptosis by binding to p53 and protecting p53 from degradation through ubiquitination by murine double minute 2 (MDM2)^4^. In addition, AIMP2 augments tumor necrosis factor-α-induced apoptotic signaling and exerts antiproliferative activities in TGF-β and Wnt pathways via distinct working mechanisms^3,5,6^. Given that these pathways are critically implicated in the control of tumorigenesis, AIMP2 is expected to act as a potent tumor suppressor with broad coverage against various types of cancer. In fact, *AIMP2* haploid mice showed increased tumor susceptibility compared to the wild-type littermates to carcinogenic treatment, confirming its tumor-suppressive activity in vivo^3^.

The full-length AIMP2 transcript consists of four exons, but a small fraction of the pre-mRNA undergoes alternative splicing to produce a variant lacking the second exon (AIMP2-DX2). AIMP2-DX2 protein compromises the tumor-suppressive activity of AIMP2 via competitive binding to p53, but fails to protect p53 from MDM2-mediated ubiquitination^7^. In contrast to AIMP2, which is mainly bound to the MSC, AIMP2-DX2 cannot work as a scaffold for MSC assembly, and thus works as a potent competitor for the tumor-suppressive activities of AIMP2^7^.

AIMP2-DX2 is receiving increasing attention as an attractive biomarker for diagnosis and prognosis^7,8^. Moreover, AIMP2-DX2 showed potential as a therapeutic target, since the downregulation of AIMP2-DX2 suppressed the growth of cancer cells and tumors in vivo^7,8^. Therefore, quantifying AIMP2-DX2 expression would allow subclassification of cancer patients and identify those who may undergo AIMP2-DX2 targeting treatment. Despite the mounting pieces of evidence, the expression of AIMP2-DX2 and its clinical implications in various types of cancer have not yet been clearly demonstrated.

The clinical application of AIMP2-DX2 has been limited due to the lack of a detection technique that allows a quantitative assessment of the AIMP2-DX2/AIMP2 expression ratio. Currently, the primary experimental approach relies on PCR amplification and examining the size difference between the two splicing variants through electrophoresis, which cannot be applied to analyze patient samples. Molecular beacon-based detection technique has been developed^9^, but its clinical applicability is questionable. Moreover, molecular beacon fails to examine both AIMP2 and AIMP2-DX2 mRNAs simultaneously in the same group of cells. Considering the competitive situation of AIMP2-DX2 and AIMP2 in carcinogenesis, simultaneous quantitation of the two variants is expected to provide a more relevant marker for accurate assessment of patient samples.

In situ hybridization (ISH) uses nucleic acid probes that are complementary to the target DNA/RNA sequences to detect and visualize the target. Clinically, DNA-ISH has been widely used to visualize DNA pathogenic variants or chromosomal structures^10^. However, as DNA does not provide information on gene expression, in particular those of alternatively spliced RNA variants, RNA-ISH is an alternative approach to investigate mRNA expressions. In addition, RNA-ISH allows analysis at a single-cell level with minimal sample disruption, which makes it an attractive clinical tool. Moreover, using multiplex single-molecule fluorescence ISH (smFISH), expression levels of both AIMP2 and its splicing variant AIMP2-DX2 mRNAs can be quantified and compared together in the same cells.

In the present study, we investigated the significance and clinical implications of AIMP2-DX2 by analyzing samples from the International Cancer Genome Consortium (ICGC) and The Cancer Genome Atlas (TCGA) database, and validated results with a clinical patient cohort of acute myeloid leukemia (AML), which was found to have the most significant association with AIMP2-DX2/AIMP2 expression ratio in terms of cancer signaling pathways. The potential of AIMP2-DX2 as a key regulator of major cancer signaling pathways in AML was further analyzed using AML cell line, which further supported our analysis of public databases. Moreover, to satisfy the unmet clinical need for a quantitative assessment of the AIMP2-DX2 expression ratio at the single-cell level, we developed an RNA-smFISH-based image analysis tool to measure AIMP2-DX2/AIMP2 expression ratio. Our image analysis showed good concordance with targeted RNA-sequencing, an alternative method to quantitate the AIMP2-DX2/AIMP2 expression ratio. Applying our image-based quantification tool, we subclassified seven hematologic cancers based on their AIMP2-DX2/AIMP2 expression ratios. More importantly, we showed that targeting AIMP2-DX2 expression in those cells with a high AIMP2-DX2/AIMP2 expression ratio could sensitize cells to anticancer drugs. To our surprise, we also found that targeting AIMP2-DX2 in cells with a low AIMP2-DX2/AIMP2 expression ratio had adverse effects and made cells more resistant to anticancer drugs. Collectively, our work provides the development of tools for quantitative assessment of alternatively spliced variants and clinical implications of AIMP2-DX2, as a potential biomarker and therapeutic target in hematologic cancer.

## Results

### Quantitation of the AIMP2-DX2 expression ratio by RNA-smFISH

**Fig. 1:**
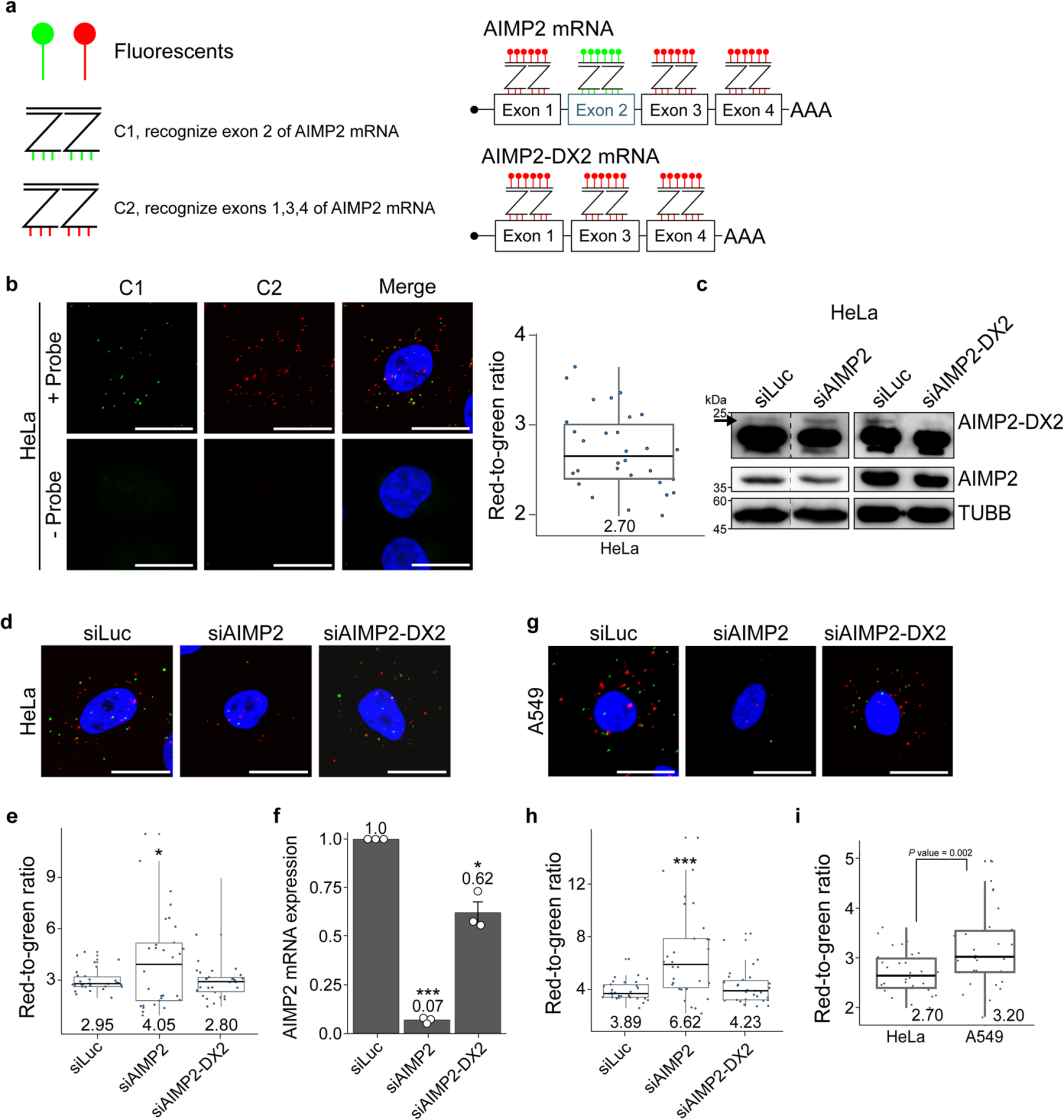
RNA-smFISH to quantify the expression ratio of AIMP2-DX2/AIMP2. **a** The design of RNA-smFISH probes. C1-tagged ZZ pairs target the exon 2 of AIMP2, and C2-tagged ZZ pairs target exons 1, 3, and 4 of AIMP2 and AIMP2-DX2. By using two different fluorophores for visualization, both probes can be hybridized and analyzed simultaneously. **b** C1 and C2 probes of the RNA-smFISH yield foci-like signal patterns in HeLa cells. Without the hybridization of probes, no fluorescent signal was detected. Bars indicate 20 μm. Box plot of the quantified red-to-green signal is shown on the right with the average ratio indicated in the bottom. Thirty images were analyzed. **c** Transfecting siRNAs specific to the full-length AIMP2 or AIMP2-DX2 effectively reduced target protein expressions in HeLa cells. The black arrow indicates the true-positive band for AIMP2-DX2. TUBB was used as loading control. **d** RNA-smFISH images upon transfecting HeLa cells with siAIMP2 or siAIMP2-DX2. Bars indicate 20 μm. **e** Box plot of RNA-smFISH red-to-green signal ratios in siRNA-transfected cells. Numbers on the box plots specify the average ratio of 30 images. **f** The knockdown efficiency of siRNAs was confirmed using RT-qPCR. The average of three replicates is shown with error bars indicating s.e.m. Numbers on the bar specify the average values. RNA expressions were measured relative to the control GAPDH. **g** RNA-smFISH images of siRNA-transfected A549 cells. Bars indicate 20 μm. **h** Box plot of red-to-green signal ratios in A549 cells. Numbers on the box plots specify the average ratio of 30 images. **i** A comparison of the red-to-green signal ratio between HeLa and A549. Numbers on the bottom indicate average ratios of 30 images. The data for HeLa is the same as the one shown in **b**. Box plot features: center line, median; box limits, upper and lower quartiles; whiskers, 1.5× interquartile range; points, individual red-to-green ratio. *P < 0.05, ***P < 0.001. For statistical analysis, a one-tailed *t* test with unequal variance was used. smFISH single-molecule fluorescence ISH.

To examine the pathophysiological implications of AIMP2 and AIMP2-DX2 in carcinogenesis, we assessed their basal mRNA expression levels using the multiplex RNAscope smFISH technique^11^. We first designed smFISH ZZ probe pairs that targeted only the exon 2 and tagged them as channel 1 (C1). We designed another set of ZZ probe pairs that recognized exons 1, 3, and 4, and tagged them as channel 2 (C2). By simultaneously hybridizing and amplifying these two sets of probes, we could visualize AIMP2 mRNAs in one color (green; C1), while another color can be designated to visualize both AIMP2 and AIMP2-DX2 mRNAs (red; C2; Fig. 1a). We tested our design using HeLa cells, and found that both C1 and C2 probes yielded foci-like signal patterns (Fig. 1b). As a control, we performed RNA-smFISH without hybridization of C1 and C2 probes, which resulted in no fluorescent signal (Fig. 1b). By quantifying and determining the red-to-green signal ratios, we can infer the relative expression ratio of AIMP2-DX2/AIMP2 mRNA variants (Fig. 1b).

To test the specificity of the probes, we first transfected HeLa cells with siRNAs that target AIMP2 or AIMP2-DX2 mRNAs, and examined the expression change using our smFISH quantification approach. Through western blotting, we confirmed the knockdown of AIMP2 and AIMP2-DX2 protein expressions (Fig. 1c). In the smFISH quantitation approach, we found that the knockdown of AIMP2 significantly increased the red-to-green ratio from 2.95 to 4.05 (*P* value = 0.022; Fig. 1d, e). Using RT-qPCR, we confirmed that the knockdown efficiency of AIMP2 was over 90%, which was also reflected in decreased AIMP2 protein expression (Fig. 1c, f). When we analyzed siAIMP2-DX2-transfected samples, the signal ratio was decreased only moderately (Fig. 1d, e). This was unexpected as C1 probes recognize only the full-length AIMP2, which should not be affected by siAIMP2-DX2 transfection. Our data imply that AIMP2 expression can be positively associated with that of AIMP-DX2, considering their competitive relationship in function. Namely, increased expression of AIMP2-DX2 can provoke the expression of AIMP2, and the reverse can also be true. In this context, siAIMP2-DX2 siRNA may downregulate both AIMP2 and AIMP2-DX2 mRNAs, which may result in a moderate change in the expression ratio of the two variants. Upon closer examination, we found a significant decrease in the fluorescent signal from the green channel, which reflected a reduction of AIMP2 expression (Fig. 1d). We further confirmed our imaging data using RT-qPCR, which showed that siAIMP2-DX2 transfection indeed decreased AIMP2 mRNAs by ~40% (Fig. 1f). This decrease in mRNA expression was also translated to a moderate reduction of AIMP2 protein expression in siAIMP2-DX2-transfected cells (Fig. 1c). Of note, quantifying the conventional PCR-based detection approach also showed a change in the expression ratio of the two variants (Supplementary Fig. 1a, b).

To examine whether our RNA-smFISH approach can be applied in cells other than HeLa, particularly for lung carcinomas where AIMP2-DX2 was reported for the first time^7^, we quantified the AIMP2-DX2/AIMP2 expression ratio in A549 lung adenocarcinoma cells (Fig. 1g, h). We again confirmed the specificity of our quantification using siRNAs (Fig. 1h). We then compared the AIMP2-DX2 expression ratios between HeLa and A549 cells. A549 cells showed a red-to-green ratio that was significantly higher than that of HeLa cells (Fig. 1i), which is consistent with the previous reports that the AIMP2-DX2 variant is frequently observed in lung carcinomas^7^. Of note, due to differences in the number of ZZ pairs between the two channels, we found that the efficiencies of the probes in capturing their target mRNAs were different. This resulted in only a few overlapping foci because there are always more red foci than the green. Consequently, we could not quantitate the absolute expression ratio of AIMP2-DX2/AIMP2 mRNAs. Nevertheless, the quantified fluorescence ratios consistently reflected changes in AIMP2 and AIMP2-DX2 mRNAs in siRNA-transfected samples. Therefore, our approach semiquantitatively reflects changes in the ratios of the two variants upon siRNA transfection and in different cellular contexts.

### Correlation of AIMP2-DX2 ratio with major cancer pathways

**Fig. 2:**
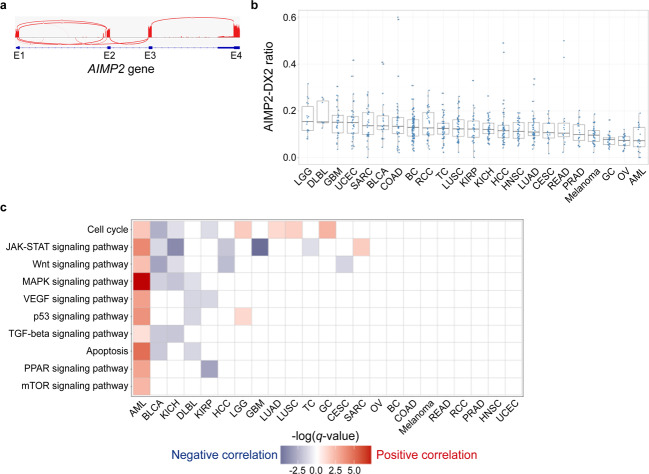
Distribution of the AIMP2-DX2/AIMP2 expression ratio and its correlation with major cancer pathways in 23 cancer types from the ICGC/TCGA. **a** An IGV sashimi plot to visualize the spliced junctions of the *AIMP2* gene. Sequencing reads spanning across exons 1–2 and exons 1–3 were considered as AIMP2 and AIMP2-DX2 mRNAs, respectively. **b** Box plot of expression ratios of AIMP2-DX2/AIMP2 from 753 cancer patients’ RNA-sequencing data on the ICGC/TCGA database plotted by cancer type. Twenty three cancer types are listed in descending order of the median AIMP2-DX2/AIMP2 expression ratio. **c** Differentially expressed gene set analysis using the GAGE method. Each tile in this figure denotes −log_10_(*q* values) of a specific pathway in each cancer type. A positive correlation is indicated by a red, while a negative correlation is denoted by blue. Box plot features: center line, median; box limits, upper and lower quartiles; whiskers, 1.5× interquartile range; points, individual AIMP2-DX2/AIMP2 expression ratio. ICGC the International Cancer Genome Consortium, TCGA The Cancer Genome Atlas, IGV Integrative Genomics Viewer, GAGE generally applicable gene-set enrichment, AML acute myeloid leukemia, BLCA bladder urothelial carcinoma, KICH kidney chromophobe, DLBL diffuse large B-cell lymphoma, KIRP kidney renal papillary cell carcinoma, HCC hepatocellular carcinoma, LGG low-grade glioma, GBM glioblastoma multiforme, LUAD lung adenocarcinoma, LUSC lung squamous cell carcinoma, TC thyroid carcinoma, GC gastric adenocarcinoma, CESC cervical squamous cell carcinoma and endocervical adenocarcinoma, SARC sarcoma, OV ovarian serous cystadenocarcinoma, BC breast invasive carcinoma, COAD colon adenocarcinoma, READ rectum adenocarcinoma, RCC kidney renal clear cell carcinoma, PRAD prostate adenocarcinoma, HNSC head and neck squamous cell carcinoma, UCEC uterine corpus endometrial carcinoma.

To test the carcinogenic potential of AIMP2-DX2 in various types of cancer, we analyzed the transcriptome data from 753 cancer patients over 23 cancer types in the ICGC/TCGA database for high-throughput analysis of AIMP2-DX2/AIMP2 expression ratios. The median age of patients from whom these samples were derived was 59 years (range 17–90 years), and 46.1% were male (Supplementary Table 1). When we visualized mapped sequencing reads on the Integrative Genomics Viewer (IGV), we could clearly observe sequencing reads corresponding to the AIMP2-DX2 mRNA, where they completely skipped the second exon (Fig. 2a). Using the ICGC and TCGA data, we directly quantified the sequencing reads spanning across exons 1–2 (AIMP2) and exons 1–3 (AIMP2-DX2), and determined the expression ratio between the two variants. We found that all types of examined cancer patients exhibited variable levels of AIMP2-DX2, where the ratio of AIMP2-DX2/AIMP2 fell into the range from 0 to 60% (Fig. 2b).

In a differentially expressed gene (DEG) set analysis, 10 out of 13 predefined major cancer pathways were shown to correlate with the AIMP2-DX2/AIMP2 expression ratio to different degrees and directions among 14 cancer types (Fig. 2c), while the other 9 cancer types (ovarian cancer, breast invasive carcinoma, colon adenocarcinoma, melanoma, rectum adenocarcinoma, kidney renal clear cell carcinoma, prostate adenocarcinoma, head and neck squamous cell carcinoma, and uterine corpus endometrial carcinoma) did not show clear correlations. Interestingly, most of the major cancer pathways in AML were highly upregulated in patients with high AIMP2-DX2/AIMP2 expression ratios (Fig. 2c). Whole differentially expressed pathways by the AIMP2-DX2/AIMP2 expression ratio are available in Supplementary Fig. 2.

### Clinical implications and the prognostic value of AIMP2-DX2

**Fig. 3:**
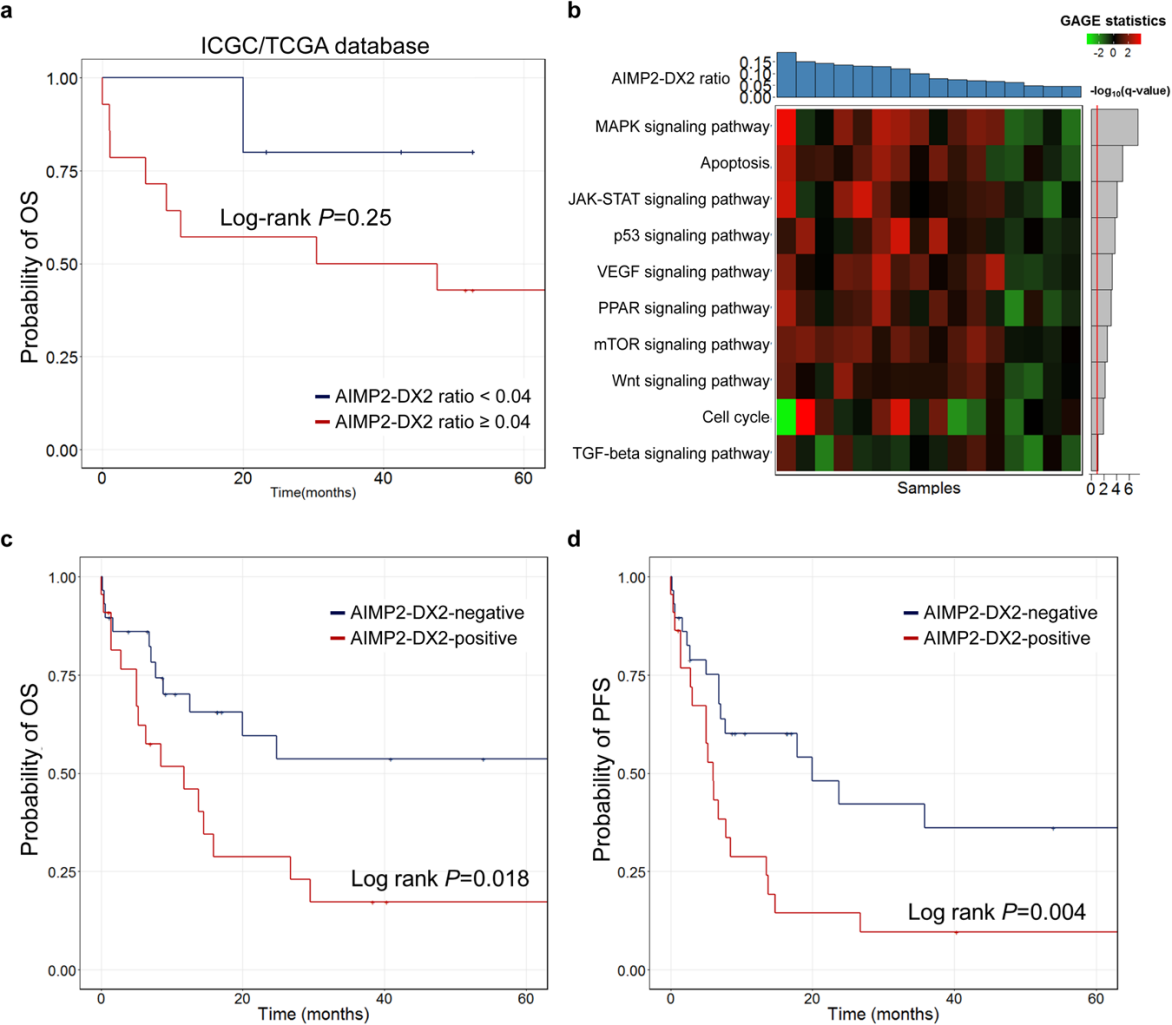
The prognostic value of AIMP2-DX2 in AML. **a** A Kaplan–Meier survival curve of OS of AML patients (*n* = 19) in the ICGC/TCGA database according to the AIMP2-DX2/AIMP2 expression ratio. The cutoff point was set to 0.04, which was the first quartile value of the AIMP2-DX2/AIMP2 expression ratio. A solid red line indicates AML samples with an AIMP2-DX2/AIMP2 expression ratio ≥0.04 (*n* = 14), while a solid blue line corresponds to those with an AIMP2-DX2/AIMP2 expression ratio <0.04 (*n* = 5). **b** A heatmap presentation of gene set perturbations of major cancer pathways for 16 AML patients having an AIMP2-DX2/AIMP2 expression ratio ≥0.04. Six AML patients having an AIMP2-DX2/AIMP2 ratio <0.04 were used as control. The two-sample *t* statistics estimated by the GAGE method are indicated by a red (upregulation) or green color (downregulation). Ten gene sets of each pathway were taken from the KEGG database. (Top) A histogram of the AIMP2-DX2/AIMP2 expression ratio of each sample in descending order. (Right) Selected gene sets ranked by −log10(*q* value). A solid red line corresponds to a −log10(*q* value) of 1.0. **c**, **d** Kaplan–Meier curves for a total of 51 AML patients stratified by AIMP2-DX2 positivity. **c** OS and **d** PFS. AIMP2-DX2 positivity was determined by RT-PCR. A solid red line indicates AML patients with AIMP2-DX2 positive (*n* = 22), while a solid blue line corresponds to those with AIMP2-DX2 negative (*n* = 29). AML acute myeloid leukemia, OS overall survival, ICGC the International Cancer Genome Consortium, TCGA The Cancer Genome Atlas, GAGE generally applicable gene-set enrichment, KEGG Kyoto Encyclopedia of Genes and Genomics, PFS progression-free survival.

We further analyzed the clinical implication of AIMP2-DX2 in hematologic cancer by analyzing AML samples from the ICGC/TCGA database. For a total of 19 AML samples, the median age of patients was 60 years (range 21–82 years), and the female to male ratio was 10:9. Using a cutoff ratio of 0.04, which was the first quartile value (*Q*_1_) of the AIMP2-DX2/AIMP2 expression ratio in AML, a Kaplan–Meier curve for the overall survival (OS) showed that patients with an AIMP2-DX2/AIMP2 expression ratio ≥*Q*_1_ tended to exhibit poor OS (median survival 47.7 months) compared to those with an AIMP2-DX2/AIMP2 expression ratio <*Q*_1_ (median survival not reached; log-rank *P* = 0.25; Fig. 3a). Of note, AIMP2-DX2 exists as a free form and is not associated with MSC^7^. Considering that most AIMP2 protein exists in MSC^12^, and only a fraction becomes dissociated to mediate stress response^4^, a small amount of AIMP2-DX2 compared to total AIMP2 (free + MSC associated) can still act as a potent inhibitor of AIMP2. Two-sample *t* statistics estimated by the generally applicable gene-set enrichment (GAGE) method for 16 AML patients with AIMP2-DX2/AIMP2 ratios ≥0.04 and 6 AML patients with AIMP2-DX2/AIMP2 ratios <0.04 are summarized in Fig. 3b, with corresponding false discovery rate (FDR) *q* values. The most differentially expressed pathway by AIMP2-DX2/AIMP2 expression ratio was the mitogen-activated protein kinase (MAPK) signaling pathway (*q* = 3.64 × 10^−8^; Fig. 3b).

To corroborate the prognostic value of AIMP2-DX2 in AML, we investigated the correlation between AIMP2-DX2 expression and survival outcomes in a clinical validation cohort of AML. For a total of 51 AML patients included in this analysis, the median age was 54.3 years (range 20.4–83.8 years; Table 1), and 23 out of 51 patients (45.1%) were female. The most common French–American–British (FAB) classification subtype was M2 (33.3%); most of the patients (64.7%) were classified into an intermediate-risk group by the Medical Research Council (MRC) criteria. Of a total of 51 AML patients, 29 patients (56.9%) were negative for AIMP2-DX2 by RT-PCR at the time of diagnosis while the other 22 patients (43.1%) were positive for AIMP2-DX2. Although no statistically significant clinical characteristics was found between the two groups, AIMP2-DX2-negative patients tended to be in a high-risk MRC group compared with AIMP2-DX2-positive patients, while the median age of AIMP2-DX2-negative patients was younger than that of AIMP2-DX2-positive patients.

**Table 1:** Clinical characteristics of AML patients in a clinical validation cohort.

Characteristics	Total (*N* = 51)	AIMP2-DX2 negative (*N* = 29)	AIMP2-DX2 positive (*N* = 22)	*P* value
Gender, *n* (%)				0.742
Male	28 (54.9)	17 (58.6)	11 (50.0)	
Female	23 (45.1)	12 (41.4)	11 (50.0)	
Age, median years (range)	54.3 (20.4–83.8)	50.8 (21.1–72.7)	61.4 (20.4–83.8)	0.213^a^
FAB classification				0.108^b^
M0	1 (2.0)	1 (3.4)	0 (0.0)	
M1	9 (17.6)	5 (17.2)	4 (13.8)	
M2	17 (33.3)	13 (44.8)	4 (13.8)	
M3	6 (11.8)	3 (10.3)	3 (10.3)	
M4	11 (21.6)	7 (24.1)	4 (13.8)	
M4e	2 (3.9)	0 (0.0)	2 (6.9)	
M5	2 (3.9)	0 (0.0)	2 (6.9)	
M7	1 (2.0)	0 (0.0)	1 (3.4)	
Not-specified	2 (3.9)	0 (0.0)	2 (6.9)	
MRC risk group				0.631^b^
Low	13 (25.5)	7 (24.1)	6 (20.7)	
Intermediate	33 (64.7)	18 (62.1)	15 (51.7)	
High	5 (9.8)	4 (13.8)	1 (3.4)	

*FAB classification* French–American–British classification, *MRC risk group* Medical Research Council risk group.

^a^Estimated by Mann–Whitney *U* test.

^b^Estimated by Fisher’s exact test.

In this cohort of AML patients, there were statistically significant differences in the OS (log-rank *P* = 0.018) and progression-free survival (PFS; log-rank *P* = 0.004) rates according to AIMP2-DX2 positivity determined by RT-PCR (Fig. 3c, d). The OS of the AIMP2-DX2-positive group (median survival 11.7 (5.03–29.6) months) was significantly inferior to that of the AIMP2-DX2-negative group (median survival not reached) with a hazard ratio (HR) of 2.47 (95% CI, 1.14–5.34; *P* = 0.022). Adjustment by age and MRC risk group gave an HR for OS of 2.48 (95% CI, 1.12–5.52; *P* = 0.026). Similarly, the AIMP2-DX2-positive group showed a worse PFS (median survival 5.97 (3.07–13.8) months) compared to the AIMP2-DX2-negative group (median survival 19.93 (7.10–not reached) months; HR, 2.59; 95% CI, 1.32–5.11; *P* = 0.006). The HR for PFS, adjusted by age, and MRC risk group, was 2.71 (95% CI, 1.35–5.45; *P* = 0.005).

The implication of the AIMP2-DX2/AIMP2 expression ratio for OS and tumor-node-metastasis stage was further investigated in other cancer types (Supplementary Fig. 3). Similar to AML, samples with an AIMP2-DX2/AIMP2 expression ratio ≥*Q*_1_ tended to exhibit an inferior OS in colon carcinoma (log-rank *P* = 0.28), and hepatocellular carcinoma (log-rank *P* = 0.24). For these cancer types, although statistically insignificant, the AIMP2-DX2/AIMP2 expression ratio showed a tendency toward increasing along with the stage. Additional studies with a larger patient number will be able to delineate clinical implications of the AIMP2-DX2/AIMP2 expression ratio in these cancers. In contrast, such a prognostic value and correlation with the stage were not evident in other cancer types. Analysis of lung adenocarcinoma is shown as an example in Supplementary Fig. 3c.

### Subclassification of hematologic cancer based on the AIMP2-DX2 ratio

The analysis of ICGC/TCGA database and the AML patient cohort result showed strong evidence for the potential role, and the clinical implication of AIMP2-DX2 in blood cancers. In fact, hematologic malignancy is cancer with a close relationship with p53 dysregulation, where AIMP2-DX2 may play a key regulatory function^13,14^. However, the current strategy to quantitate AIMP2-DX2/AIMP2 expression ratio relies on RT-PCR, which is prone to high false-positive rates due to analysis at the population level. In particular, even normal cells can express a low level of AIMP2-DX2, indicating that the percentage of cancer cells in the population may affect the expression ratio of AIMP2-DX2/AIMP2. We turned to imaged-based RNA-smFISH for increased accuracy in quantifying the AIMP2-DX2/AIMP2 expression ratio, as this approach allows analysis at the single-cell level.

**Fig. 4:**
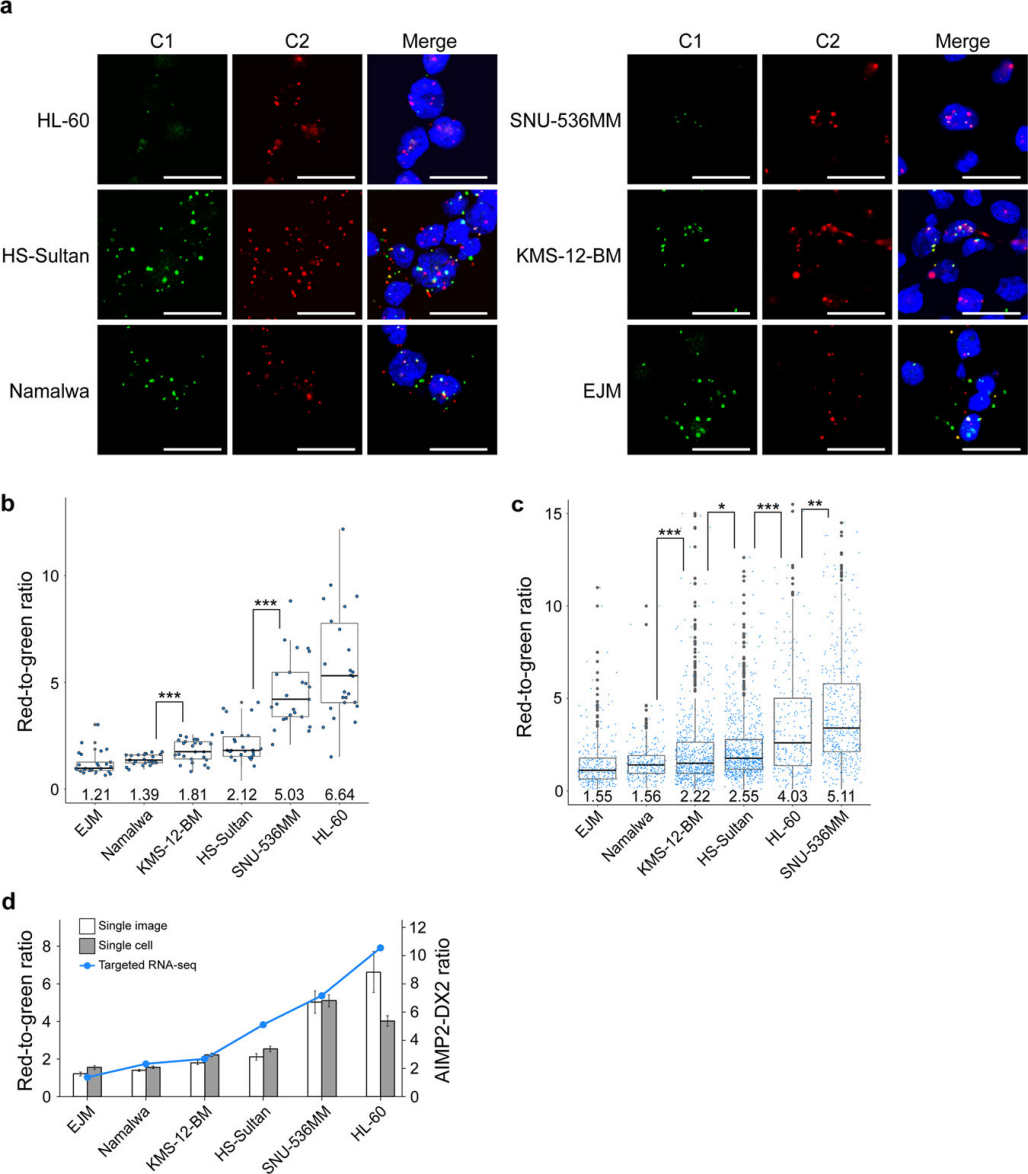
RNA-smFISH to subclassify hematologic cancer cells. **a** RNA-smFISH images of six hematologic cancer cell lines. Bars indicate 20 μm. **b** Box plot of the quantified red-to-green signal ratio of six hematologic cancer cell lines. One ratio was calculated per image. Numbers on the box plots specify the average ratios. *n* = 25 for EJM, Namalwa, KMS-12-BM, and SNU-536MM. *n* = 26 for HS-Sultan and HL-60. **c** Box plot of RNA-smFISH single-cell analysis of six hematologic cancer cell lines. Numbers on the box plots specify the average ratios. *n* = 287 for EJM, *n* = 273 for Namalwa, *n* = 665 for KMS-12-BM, *n* = 737 for HS-Sultan, *n* = 299 for HL-60, and *n* = 468 for SNU-536MM cells. **d** Comparison of red-to-green ratios of smFISH image quantitation for single-image (white bar) and single-cell (gray bar) analysis, and AIMP2-DX2 ratio of targeted RNA-sequencing (blue line). Bar graphs and the point plot indicate the mean of the corresponding method. Box plot features: center line, median; box limits, upper and lower quartiles; whiskers, 1.5× interquartile range; points, individual red-to-green ratio. *P < 0.05, **P < 0.01, ***P < 0.001. For statistical analysis, a one-tailed *t* test with unequal variance was used. smFISH single-molecule fluorescence ISH.

To establish smFISH as a subclassification tool in hematologic cancer, we performed RNA-smFISH and quantitated expression ratios of AIMP2-DX2/AIMP2 in six different blood cancer cell lines. Due to the round shape of the cells, we observed fewer foci per cell, but the size of foci was larger than those of adherent cells examined above (Fig. 4a). Our analysis revealed that the expression ratio was variable across different blood cancer cell lines, but more uniform within a single-cell line (Fig. 4a, b). HL-60 acute promyelocytic leukemia cells showed the highest expression ratio, while EJM multiple myeloma cells showed the lowest value (Fig. 4b). Of note, for the quantification, we performed analysis per image at this stage. Each data point in Fig. 4b indicates the red-to-green ratio per image, which is an average of ~18 cells.

One advantage of our approach is that it allows image-based analysis at the single-cell level. We first identified individual cells based on the DAPI nuclei counterstain. We then assigned red and green signals to a cell based on the distance to the closest DAPI signal. Once the fluorescent signals were assigned, we determined the red-to-green signal ratio for individual cells. The single-cell analysis resulted in increased variation due to cell-to-cell variabilities, but the overall subclassification remained the same (Fig. 4c). Of note, we also performed the single-cell analysis on adherent cells (HeLa and A549) and again obtained the same conclusion that the expression ratio of AIMP2-DX2/AIMP2 is higher in A549 cells (Supplementary Fig. 4).

To validate the AIMP2-DX2 expression patterns, we performed targeted RNA-sequencing and compared the sequencing result with that of RNA-smFISH image analyses. We analyzed 16 human cancer cell lines (Supplementary Table 2), and seven (six blood cancer cell lines and A549) of which were also used in the RNA-smFISH analysis. The expression ratios of AIMP2-DX2/AIMP2 were calculated using the same approach as our analysis of the ICGC/TCGA data. A comparison of RNA-smFISH single-image quantitation, single-cell analysis, and targeted RNA-sequencing is summarized in Fig. 4d. We found that relative ratios of the two variants in these six blood cancer cell lines showed good agreement with that of the RNA-smFISH analysis (Fig. 4d). Collectively, these results show that RNA-smFISH image analysis can be a reliable tool to quantitatively analyze and subclassify cells based on their expression ratio of AIMP2-DX2/AIMP2.

### Drug sensitization by modulating AIMP2-DX2 expression

**Fig. 5:**
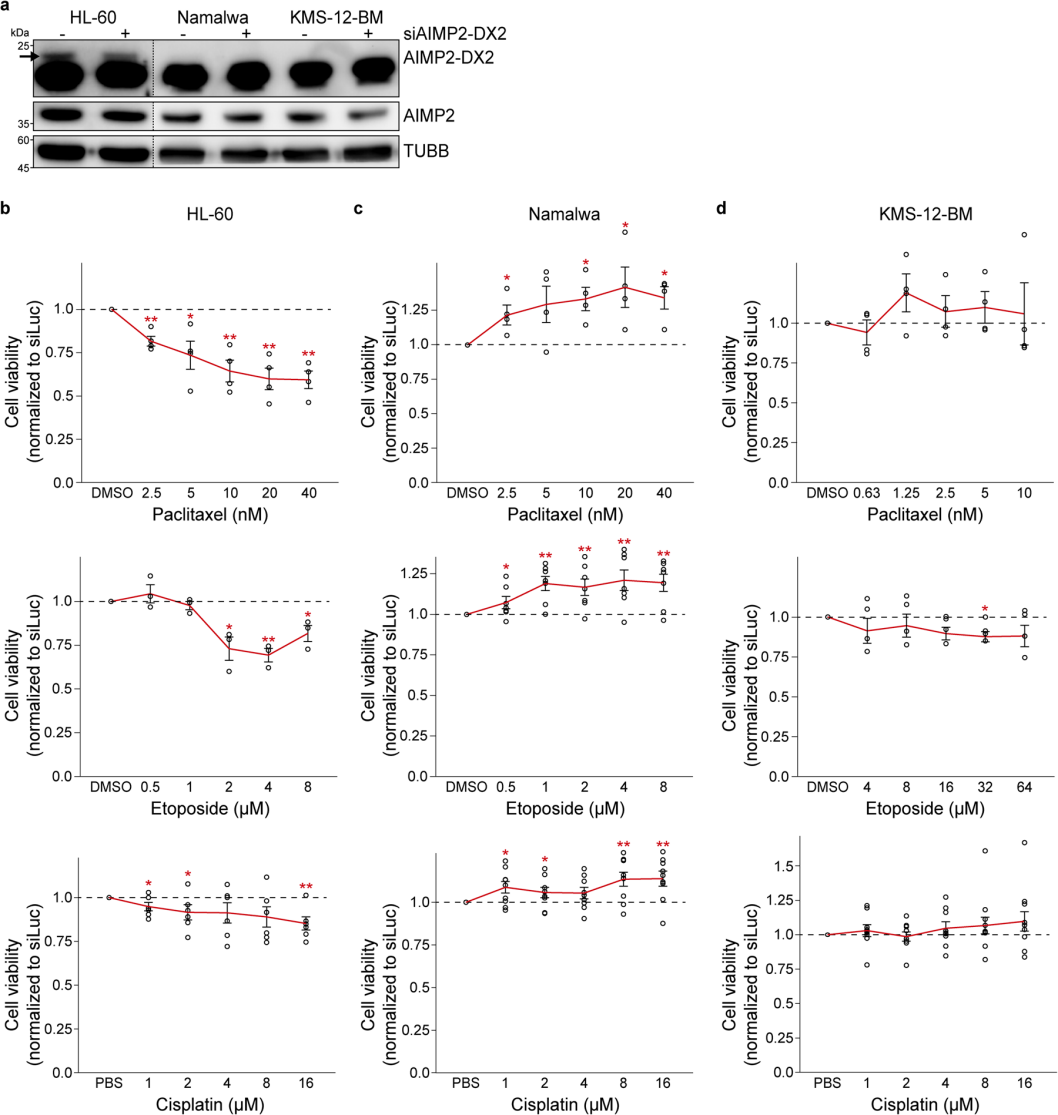
Targeting AIMP2-DX2 expression can sensitize cells to anticancer drugs. **a** AIMP2-DX2 protein expression after electroporation with siRNA targeting AIMP2-DX2 mRNA. AIMP2-DX2 protein was detected only in HL-60, but not in Namalwa nor KMS-12-BM cells. In HL-60 cells, siAIMP2-DX2 effectively reduced the AIMP2-DX2 protein expression. TUBB was used as loading control. **b**–**d** Knockdown of AIMP2-DX2 enhanced apoptosis induced by paclitaxel, etoposide, and cisplatin in HL-60 cells (**b**), but not in Namalwa (**c**) nor KMS-12-BM cells (**d**). For Namalwa cells, knockdown of AIMP2-DX2 partially rescued the cell death by all drugs. *n* = 4 for paclitaxel in all three cell lines. *n* = 3 for HL-60, *n* = 7 for Namalwa, *n* = 4 for KMS-12-BM for etoposide. *n* = 6 for HL-60, *n* = 9 for Namalwa, *n* = 9 for KMS-12-BM for cisplatin. A solid red line indicates cell viability relative to siLuc, while a black dashed line indicates cell viability of control. For all data presented, an average is shown with error bars indicate s.e.m. *P < 0.05, **P < 0.01. For statistical analysis, a one-tailed *t* test with unequal variance was used.

Considering the working mechanism of AIMP2-DX2, that it inhibits the protective activity of AIMP2 on p53 during stress, we investigated whether targeting AIMP2-DX2 expression can sensitize cells to anticancer drugs. More importantly, we asked whether such sensitization depends on the expression ratio of AIMP2-DX2/AIMP2. For those cells with a low ratio, targeting AIMP2-DX2 should not have any effect, while cells with a high ratio should result in increased cell death. To target AIMP2-DX2, we performed electroporation with siAIMP2-DX2 to three blood cancer cell lines. HL-60 promyelocytic leukemia cells, which expressed the highest ratio of AIMP2-DX2/AIMP2, clearly showed decreased expression of AIMP2-DX2 (Fig. 5a). The expression of AIMP2-DX2 in the other two cell lines was too low to be detectable. However, we optimized the electroporation condition using a control target and applied the same condition to transfect siAIMP2-DX2.

We treated a low dose of paclitaxel to HL-60 and compared the drug sensitivity after downregulating AIMP2-DX2 (Fig. 5b and Supplementary Fig. 5a). We chose paclitaxel as an anticancer drug of interest because apoptosis induced by a low-dose treatment of paclitaxel is associated with normal p53 expression^15,16^. In our experiment, we asked whether sensitivity to paclitaxel could be enhanced by targeting AIMP2-DX2 in hematologic cancer. More importantly, we examined whether such effect depends on the expression ratio of AIMP2-DX2/AIMP2 quantified by RNA-smFISH. Consistent with our expectation, knockdown of AIMP2-DX2 significantly decreased cell viability upon paclitaxel treatment in HL-60 cells (Fig. 5b). As a comparison, we used Namalwa Burkitt’s lymphoma cells, which expressed a low ratio of AIMP2-DX2/AIMP2. Unexpectedly, knockdown of AIMP2-DX2 expression resulted in increased cell viability upon paclitaxel treatment (Fig. 5c). One possibility is the positive association between AIMP2 and AIMP2-DX2, as we found earlier (Fig. 1d, f). In this context, downregulating AIMP2-DX2 resulted in a significant decrease in the full-length AIMP2 expression, which compromised normal p53 function. Consequently, cells with low AIMP2-DX2 expression showed adverse effects to paclitaxel treatment.

Lastly, we used KMS-12-BM myeloma cells, which expressed the intermediate AIMP2-DX2/AIMP2 expression ratio. In this case, knockdown of AIMP2-DX2 did not show a significant effect on drug sensitivity, suggesting that targeting AIMP2-DX2 may not provide any benefit for the intermediate expression group (Fig. 5d). Our results indicate that targeting AIMP2-DX2 expression can be an effective clinical strategy to enhance the efficacy of paclitaxel. Furthermore, prior quantification of AIMP2-DX2/AIMP2 expression ratio appears to be critical as targeting AIMP2-DX2 expression in cells with a low AIMP2-DX2/AIMP2 expression ratio can provoke adverse effect, as in the case of Namalwa cells.

We performed an analogous set of experiments, but this time using etoposide or cisplatin instead of paclitaxel to trigger apoptosis. We chose etoposide because it induces DNA damage and initiates p53 signaling^17,18^. In addition, cisplatin is a p53-dependent anticancer drug^19,20^, currently being used to treat refractory lymphomas and AML^21–24^. We found that only the high AIMP2-DX2-expressing HL-60 cells could be sensitized to etoposide and cisplatin via knockdown of AIMP2-DX2 (Fig. 5b). At the same time, transfecting Namalwa cells with siAIMP2-DX2 again showed the adverse effect, where it partially rescued cell death by etoposide and cisplatin (Fig. 5c). Lastly, targeting AIMP2-DX2 did not have any effect on apoptotic response in KMS-12-BM cells (Fig. 5d). Together, our results clearly indicate that targeting AIMP2-DX2 expression can be used to enhance the effectiveness of anticancer drugs, such as paclitaxel, etoposide, and cisplatin that rely on the p53 signaling pathway.

### Targeting AIMP2-DX2 in AML affects major cancer pathways

**Fig. 6:**
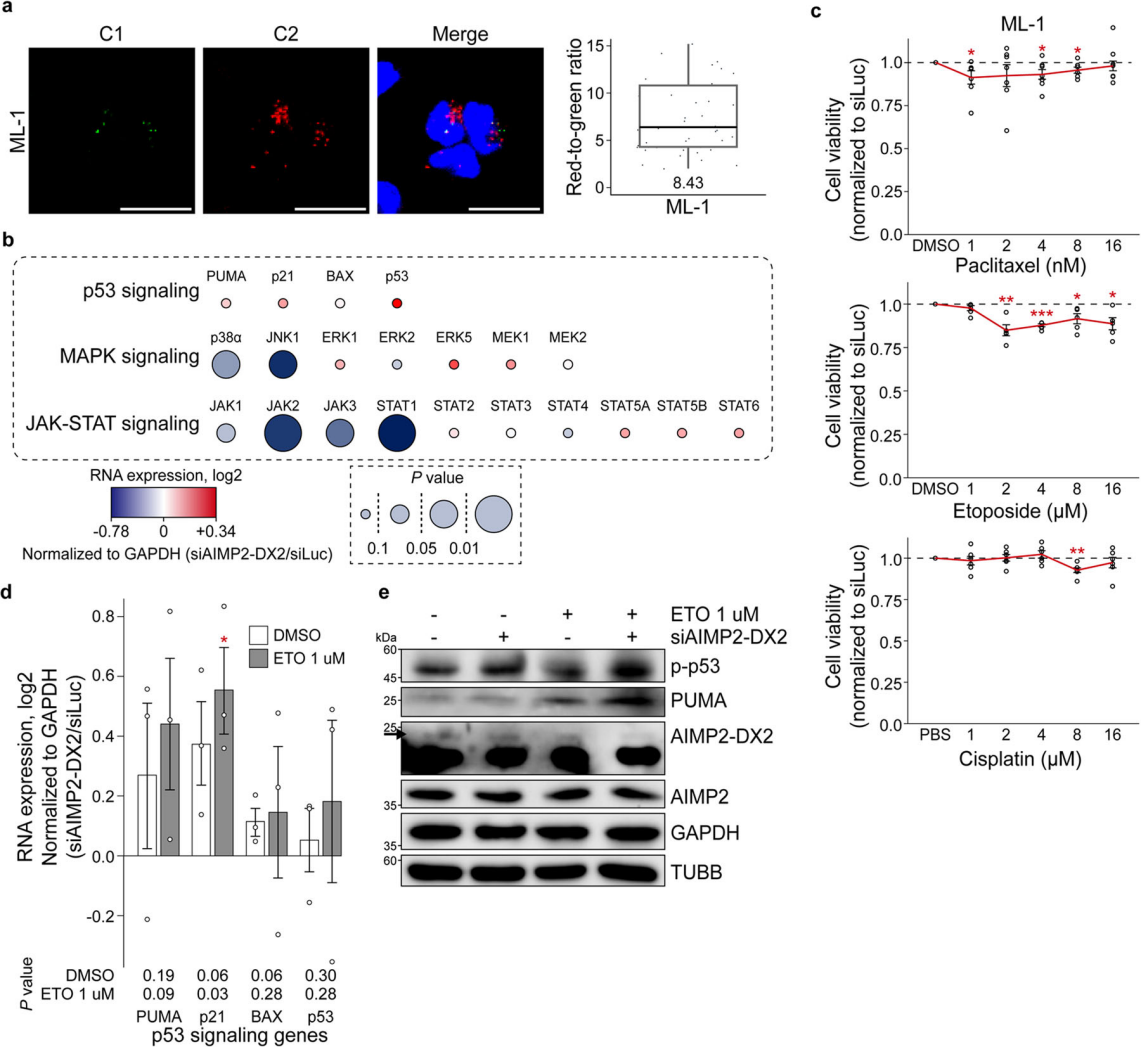
Analysis of AIMP2-DX2 in AML. **a** RNA-smFISH images of ML-1 cells. Bars indicate 20 μm. Box plot of the quantified red-to-green signal is shown on the right with the number in the bottom denotes the average ratio of 51 images. **b** AIMP2-DX2 is positively correlated with MAPK and JAK-STAT signaling pathways in ML-1 cells. The RNA expression profiles of representative genes for p53, MAPK, and JAK-STAT signaling pathways are visualized. GAPDH normalized fold change and its *P* values are indicated as color and size of the corresponding circle, respectively. *n* = 6 for p53 signaling pathway genes, and *n* = 7 for other signaling pathway genes. **c** Electroporation with siAIMP2-DX2 enhances apoptosis induced by paclitaxel, etoposide, and cisplatin in ML-1 cells. *n* = 7 for paclitaxel, *n* = 5 for etoposide, *n* = 6 for cisplatin. A solid red line indicates cell viability relative to siLuc, while a black dashed line indicates cell viability of control. For all data presented, the average is shown with error bars indicate s.e.m. **d**, **e** Knockdown of AIMP2-DX2 can activate the p53 signaling pathway upon etoposide treatment. In **d**, the RNA expression of the representative genes for the p53 signaling pathway is examined. For all data presented, an average of three biological replicates is shown with error bars indicate s.e.m. The numbers under the graph denote the *P* values. In **e**, protein expression of phosphorylated p53 and PUMA are examined via western blotting. The black arrow indicates the band for the AIMP2-DX2 protein. GAPDH and TUBB were used as loading control. Box plot features: center line, median; box limits, upper and lower quartiles; whiskers, 1.5× interquartile range; points, individual red-to-green ratio. *P<0.05, **P<0.01. For statistical analysis, a one-tailed *t* test with unequal variance was used. AML acute myeloid leukemia, smFISH single-molecule fluorescence ISH, ETO etoposide.

Considering the ICGC/TCGA analysis that several cancer pathways showed a positive correlation with the ratio of AIMP2-DX2/AIMP2 in AML, we extended our analysis using ML-1 AML cells. First, we applied the RNA-smFISH approach to quantitate the AIMP2-DX2/AIMP2 expression ratio in ML-1 cells. We found that the red-to-green ratio has a mean value of 8.43, which categorizes ML-1 to a high expression group (Fig. 6a). This is consistent with the targeted RNA-sequencing result that ML-1 is one of the cell lines with a high expression ratio of AIMP2-DX2/AIMP2 (Supplementary Table 2). Next, we utilized these cells to verify the result of the ICGC/TCGA database analysis that AIMP2-DX2/AIMP2 expression ratio correlates with major cancer signaling pathways in AML. We knocked down the expression of AIMP2-DX2 by transfecting siAIMP2-DX2 and examined its effect on components of p53, MAPK, and JAK-STAT signaling pathways using RT-qPCR. Among the genes examined, p38α and c-Jun N-terminal kinase 1 (JNK1) of MAPK signaling and JAK1-3 and STAT1 of JAK-STAT signaling showed a positive correlation with the AIMP2-DX2 expression (Fig. 6b). This is consistent with our analysis of the ICGC/TCGA database that AIMP2-DX2 is positively correlated with MAPK and JAK-STAT signaling pathways in AML (Figs. 2c and 3b). On the contrary, we could not find a correlation between p53 signaling pathway and AIMP2-DX2 expression. Nevertheless, our data suggest that AIMP2-DX2 may regulate tumorigenesis in AML.

We further investigated whether targeting AIMP2-DX2 can sensitize cells to anticancer drugs. Considering that ML-1 belongs to a high expression ratio group, downregulating AIMP2-DX2 should sensitize cells to the drug treatment. Indeed, similar to the case of HL-60, knockdown of AIMP2-DX2 resulted in increased sensitivity to paclitaxel, etoposide, and, to a limited degree, cisplatin (Fig. 6c and Supplementary Fig. 6). We then examined the activation of the p53 signaling pathway when cells were treated with etoposide, using qPCR and western blotting. Although our earlier analysis showed no correlation between p53 signaling and AIMP2-DX2 without any stressor (Fig. 6b), when we treated etoposide, p21 and PUMA mRNAs were strongly induced in AIMP2-DX2-deficient cells (Fig. 6d). Moreover, we found increased phosphorylation of p53 and increased expression of PUMA protein when etoposide was treated in AIMP2-DX2 knockdown cells (Fig. 6e). Collectively, these data support that the downregulation of AIMP2-DX2 in ML-1 cells can affect their response to anticancer drugs by modulating p53 signaling pathway.

## Discussion

In this study, we developed an RNA-smFISH image analysis tool to quantify the expression ratio of tumorigenic alternatively spliced AIMP2-DX2. Together with the analysis of the public database, we found that AIMP2-DX2 was universally expressed to varying degrees among diverse cancers, and that the AIMP2-DX2/AIMP2 expression ratio estimated by RNA-smFISH could be reliable indicators of AIMP2-DX2 expression. Of 23 types of cancer in the ICGC/TCGA database, AIMP2-DX2/AIMP2 expression ratio was most strongly correlated with major cancer pathways, such as the MAPK signaling pathway, in AML. More importantly, subclassification of hematologic cancers based on their AIMP2-DX2/AIMP2 expression ratios can be used to design patient-specific treatment strategies. As a proof of concept, we showed that targeting AIMP2-DX2 sensitized cells to paclitaxel, etoposide, and cisplatin treatments only in those cells with a high AIMP2-DX2/AIMP2 expression ratio. Moreover, the expression ratio of AIMP2-DX2 also had prognostic value in AML.

AML is ranked among the most lethal of hematological malignancies. Despite extensive efforts that have been put forward into understanding the pathogenesis of AML, the outcome for adult patients with AML remains poor in the form of a high mortality rate^25^. Therefore, better knowledge of the disease’s pathology, as well as novel therapeutic strategies, are exigent. In the present study, AIMP2-DX2 expression in AML positively correlated with most major cancer pathways, which have crucial roles in tumorigenesis and are suggested to be potential targets in AML patients^26^. In addition, patients with positive AIMP2-DX2 expression exhibited inferior survival outcomes. These results indicate that AIMP2-DX2 could be a potential biomarker of AML and a novel therapeutic target.

The efficacy of targeting AIMP2-DX2 for controlling tumorigenesis has already been reported in lung and ovarian cancers, using both in vivo and in vitro models. As a result, several novel pharmaceuticals having such activity are currently under development. For example, a small chemical compound called BC-DXI01 showed specific inhibitory activity in AIMP2-DX2-positive lung cancer cell lines by selectively suppressing AIMP2-DX2 mRNA^27^. Similarly, SLCB050, a novel chemical compound that blocks the interaction between AIMP2-DX2 and p14ARF, reduced the viability of lung cancer cells^28^. Moreover, a trans-splicing ribozyme targeting AIMP2-DX2 mRNA showed specific and effective retardation of lung cancer^29^. In this regard, suppressing AIMP2-DX2 expression could theoretically be a novel therapeutic strategy for AML as well. Indeed, we showed that downregulating AIMP2-DX2 expression in ML-1 AML cells resulted in increased cell death by paclitaxel, etoposide, and to a certain degree, cisplatin. At the same time, targeting AIMP2-DX2 can result in adverse effects in a subgroup of cells with a low AIMP2-DX2/AIMP2 expression ratio, most likely due to the positive association between AIMP2 and AIMP2-DX2. Thus, discovering novel drugs targeting AIMP2-DX2 and determining its working mechanism at the molecular level would likely contribute to an improvement in the treatment of AML and other hematologic cancer patients.

According to our analysis, AIMP2-DX2 expression ratio is positively correlated with MAPK and JAK-STAT signaling pathways in AML. This is consistent with previous studies that showed the importance of these pathways in AML. It has been shown that JNK contributes to drug resistance and hyperleukocytosis in AML^30^. In addition, p38 MAPK positively regulates proliferation in hematopoietic cells^31^. Moreover, activated JAKs phosphorylate STAT proteins which results in their dimerization and translocalization into the nucleus to transcribe genes involved in cell survival and proliferation^32^. One study reported elevated levels of p-JAK2 in bone marrow samples from patients with AML (*n* = 77) and its correlation to shorter survival in patients, with either de novo or secondary AML^32,33^. Indeed, treating AML cells with AZ960, a JAK2 inhibitor, induced apoptosis, indicating the importance of JAK-STAT signaling. In the future, investigation on the regulation of MAPK and JAK-STAT signaling systems by AIMP2-DX2 may provide a better understanding of tumorigenesis of AML.

Considering the tumorigenic activity of AIMP2-DX2 and its universal expression, the potential of AIMP2-DX2 as a biomarker would not be limited to AML. For colon adenocarcinoma and hepatocellular carcinoma in the ICGC/TCGA database, although statistical significance was not met, patients with an AIMP2-DX2/AIMP2 expression ratio ≥*Q*_1_ displayed worse survival outcomes (Supplementary Fig. 3). On the other hand, such a tendency was not observed in lung cancer patients. However, considering the retrospective nature of clinical information in the ICGC/TCGA database, this result should be interpreted with caution. In addition, the application of *Q*_1_ as a cutoff value in lung cancer may bias this result because the optimal cutoff AIMP2-DX2/AIMP2 expression ratio may differ for each cancer type. Indeed, a previous report demonstrated that AIMP2-DX2 inversely correlated with the survival of lung cancer patients^7^. Therefore, further studies validating the prognostic significance of AIMP2-DX2 and optimizing its cutoff value in various cancers are necessary.

The presence of AIMP2-DX2 provides important insights into tumorigenesis and the progression of cancers. However, detailed studies have not been reported concerning how to detect AIMP2-DX2. Although direct sequencing has been widely used for detecting mutations, it is labor-intensive and time-consuming for routine clinical analysis^34^. In the present study, we developed an image-based analysis that can quantitate the AIMP2-DX2/AIMP2 mRNA expression ratio at the single-cell level. In addition, targeted RNA-sequencing was applied for quantifying levels of AIMP2 and AIMP2-DX2 mRNAs. Together, these methods provide a rapid and highly accurate approach for quantifying the expression ratio of AIMP2-DX2/AIMP2. However, because almost every cancer patient displayed a nonzero AIMP2-DX2/AIMP2 expression ratio, the optimal cutoff value needs to be determined for defining AIMP2-DX2 positivity, as is the case for the her2/cep17 ratio in breast cancer^35^. With a comprehensive understanding of the role of AIMP2-DX2, various diagnostic methods for detecting AIMP2-DX2 are currently under the development.

## Methods

### Accessing the ICGC/TCGA database and file processing

To analyze the distribution and clinical implication of AIMP2-DX2/AIMP2 expression ratio of samples in the ICGC/TCGA database, clinical data with a corresponding RNA-sequencing bam file aligned by Tophat2^36^ were downloaded from the Cancer Genomics Hub of the sequencing programs of the National Cancer Institute, using GeneTorrent^37^. Expression levels of AIMP2 and AIMP2-DX2 mRNAs for each sample were estimated as described in the targeted RNA-sequencing section. HTSeq-count, open-source software for the analysis of high-throughput sequencing data, was used to count whole RNA-sequencing reads for differential expression analysis, as described in the next section^38^.

### Differentially expressed gene set analysis

After processing with HTSeq-count, the RNA-sequencing data of the ICGC/TCGA database were analyzed for DEG set analysis. DEGs by AIMP2-DX2/AIMP2 expression ratio were identified using an R package, *DESeq2*^39^, and then were utilized to identify differentially regulated pathways (or gene sets) using another R package, *gage*^40^. Candidate genes involved in each canonical pathway were taken from the KEGG (Kyoto Encyclopedia of Genes and Genomics) pathway database (Release 79.1, September 2016)^41^. Among various pathways, the analysis was focused on the ten major cancer pathways.

### Statistics and reproducibility

The negative binomial generalized linear model was applied for identifying genes differentially expressed by AIMP2-DX2/AIMP2 expression ratio^39^. DEG set analysis was carried out by using the GAGE method^40^, and the test statistics across samples were summarized at a pathway level with an FDR *q* value. Because tumorigenesis is controlled by AIMP2 in a dosage-dependent manner^3^, and AIMP2-DX2 competitively inhibits the activity of AIMP2, the AIMP2-DX2/AIMP2 expression ratio was assumed to affect the expression of gene sets in a dose-dependent manner. Accordingly, the AIMP2-DX2/AIMP2 expression ratio was considered as a continuous variable in the DEG set analysis. Differences in continuous variables between two groups in a clinical validation cohort were analyzed by the unpaired *t* test or the Mann–Whitney *U* test, if appropriate. Survival analysis was performed using the Kaplan–Meier method and compared using a log-rank test. The Cox proportional hazard regression model was applied to determine the HR for AIMP2-DX2 positivity with respect to OS and PFS. For all statistical analyses, *P* values < 0.05 or FDR *q* values < 0.10 were considered statistically significant. All statistical analyses were carried out using R version 3.3.1 (http://www.r-project.org). For cell studies, statistical analysis was performed with the Microsoft Excel 2016 software. Statistical significance was determined by using one-tailed *t* test with unequal variance. All data are presented as mean ± s.e.m. as indicated in the figure legends. *P* value < 0.05 was considered as statistically significant (*P < 0.05, **P < 0.01, ***P < 0.001). The sample size *n* is presented in the figure legends.

### Analysis of the AML clinical patient cohort

A total of 51 adult AML patients, who agreed to donate bone marrow samples for research after giving informed consent, were included in the clinical validation cohort. Bone marrow samples of these patients at the time of diagnosis were collected using PAXgene Blood RNA tubes (PreAnalytiX, Hombrechtikon, Switzerland). The AIMP2 and AIMP2-DX2 mRNA levels were assessed by RT-PCR. Patients were divided into two groups according to the positivity of AIMP2-DX2. The diagnosis of AML was based on the WHO criteria, and AML was classified according to the FAB classification system. Patients were stratified into three risk groups based on cytogenetic and molecular analyses of bone marrow samples according to the refined MRC criteria^42^. Data regarding patient demographics and survival outcomes were obtained by a review of medical records. OS was defined as the duration from diagnosis to death from any cause, and PFS was defined as the time from diagnosis until relapse or death from any cause. Patients who were alive were censored at the date of the last contact. This protocol was approved by the Seoul National University Hospital Institutional Review Board (IRB approval number: 1201-099-396). This study was conducted in accordance with the Declaration of Helsinki provisions.

### RT-PCR for detecting AIMP2 and AIMP2-DX2 expression

The cDNA EcoDry Premix (Clontech laboratories, Mountain View, CA, USA) was used to convert RNAs to cDNAs, following the manufacturer’s recommendations. The primer pairs were designed for full-length AIMP2 and AIMP2-DX2, separately. For full-length AIMP2, the forward primer was designed to target the splicing junction between exons 1 and 2, while the forward primer for AIMP2-DX2 was designed to target the splicing junction between exons 1 and 3, skipping exon 2. A common reverse primer targeting exon 3 of the AIMP2 was used. In addition, one more primer pair was designed to target exons 1 and 3 to amplify both full-length AIMP2 (1078 bp) and AIMP2-DX2 (871 bp). Primer sequences for RT-PCR used in this study are provided in Supplementary Table 3.

### Targeted RNA-sequencing for AIMP2 and AIMP2-DX2

Targeted RNA-sequencing was performed using the Ion AmpliSeq targeting the *AIMP2* gene. Library construction was performed using the Ion AmpliSeq Library Kit 2.0 (Life Technologies, Waltham, MA, USA), and library templates were prepared and barcoded for sequencing using the Ion OneTouch System, as per manufacturer’s instructions. Sequencing reads were processed using the Ion Torrent Suite Software v 4.0.2 (Life Technologies). Demultiplexed samples were assessed for sequencing quality, and high-quality reads were mapped to the complete hg19 human genome (UCSC version, February 2009). Expression levels of full-length AIMP2 and AIMP2-DX2 mRNAs were estimated by samtools^43^. Reads having junctions between exons 1 and 2 (chr7:6009499-6015145) were defined as the full-length AIMP2, while those having junctions between exons 1 and 3 (chr7:6009499-6017814) were defined as AIMP2-DX2. In terms of a CIGAR string, reads corresponding to AIMP2-DX2 had a longer length of skipped lesion than those of wild-type AIMP2.

### Cell lines

Seventeen cancer cell lines were cultured under recommended conditions (Supplementary Table 4) in a 37 °C and 5% CO_2_ humidified incubator. Cell lines were purchased from American Type Culture Collection (ATCC; Manassas, VA, USA), Deutsche Sammlung von Mikroorganismen und Zellkulturen (GmbH, Braunschweig, Germany), and Korean Cell Line Bank (Seoul, Korea). SNU-536MM was internally established at Seoul National University Hospital using a bone marrow sample of multiple myeloma patients. Each culture media (Welgene Inc., Daegu, Korea) was supplemented with heat-inactivated fetal bovine serum, penicillin/streptomycin, sodium pyruvate, and L-glutamine (GIBCO; Grand Island, NY, USA). All of the suspension and adherent cells were cultured and stabilized at least for 24 h before using them for experiments. All cell lines were authenticated by short tandem repeat (STR) profiling and were tested regularly for mycoplasma contamination. According to the International Cell Line Authentication Committee register (https://iclac.org/), cross contamination has been reported in HS-Sultan cells where no authentic stock is known. We used HS-Sultan cell line authenticated by ATCC, using STR profiling analysis.

### Sample preparation and RNA extraction

In order to assess the plausibility of an AIMP2-DX2/AIMP2 expression ratio for determining AIMP2-DX2 positivity, targeted RNA-sequencing was performed in 16 cancer cell lines (Supplementary Table 2). The RNAs of these cancer cell lines were isolated using TRIzol reagent (Life Technologies, Waltham, MA, USA) with 1 mL TRIzol, 200 μL chloroform, 500 μL isopropanol, and 1 mL 75% ethanol in accordance with manufacturer’s instructions.

### Nucleic acid probes

ISH probes for AIMP2 were designed and purchased from Advanced Cell Diagnostics. RNAscope probe Hs-AIMP2-E2 (cat. no. 444981) targets exon 2 of AIMP2, while Hs-AIMP2-E1E3E4 (cat. no. 451471) targets exons 1, 3, and 4 of AIMP2 and AIMP2-DX2 mRNAs. These two probes were labeled with C1 and C2, respectively, and the multiplex detection kit was used to visualize them simultaneously.

### RNA-smFISH

RNA-smFISH was performed according to the RNAscope Multiplex Fluorescent Assay (ACD, Inc., Hayward, California, USA) protocol with an additional step to attach suspension cells on to a microscope slide. Cells were washed with phosphate-buffered saline (PBS) and fixed in 4% paraformaldehyde (Electron Microscopy Sciences, Hatfield, PA, USA) for 1 h at room temperature. Fixed cells were washed two times with PBS, and resuspended with 70% EtOH and kept overnight at 4 °C to permeabilize. Cells were spread on a microscope slide and attached on to the slide by drying it at room temperature for 40 min. This step was omitted when adherent cells were used. Cells were then dehydrated by a subsequent wash with 50, 70, and 100% EtOH. Dehydrated cells were treated with Protease K (RNAscope® pre-treat kit, cat. No.322330) for 30 min at 40 °C. Cells were then hybridized with AIMP2 probes (cat. no. 444981 and 451474) for 2 h at 40 °C. Subsequent signal amplification steps were performed by treating cells with AMP1-FL, AMP2-FL, AMP3-FL, and AMP4-FL reagents of the RNAscope detection kit (cat. no. 323382). Cells were incubated in the DAPI solution for 1 min at room temperature to counterstain the nuclei. Mounting solution (Vector Labs, California, USA) was applied, and Zeiss confocal microscope (LSM780; Carl Zeiss Microscopy, GmbH, Germany) with a 40× objective (NA = 1.20) was used for imaging.

### RNA-smFISH image analysis

RNA-smFISH images were analyzed with a program coded using MATLAB (Math Works, Natick, MA, USA) v2014b image processing tool. Images were split into two single color channels (red and green) using an intensity threshold, and the two-channel images were analyzed simultaneously. The noise was identified based on the object size and removed from the image. Individual objects from the two channels were identified, and their pixel areas were measured. The ratio of red/green, which reflects the expression ratio of AIMP2-DX2/AIMP2, was calculated by dividing the average pixel areas from the red channel to that of the green channel, as shown below:

$$\frac{{{\mathrm{Red}}}}{{{\mathrm{Green}}}} = \frac{{\alpha \left( {\left[ {{\mathrm{AIMP}}2} \right] + [{\mathrm{AIMP}}2 - {\mathrm{DX}}2]} \right)}}{{\beta \left( {[{\mathrm{AIMP}}2]} \right)}} = \frac{\alpha }{\beta }\left( {1 + \frac{{[{\mathrm{AIMP}}2 - {\mathrm{DX}}2]}}{{[{\mathrm{AIMP}}2]}}} \right),$$

$$\frac{{[{\mathrm{AIMP}}2 - {\mathrm{DX}}2]}}{{[{\mathrm{AIMP}}2]}} = \frac{{{\mathrm{Red}}}}{{{\mathrm{Green}}}}\frac{\beta }{\alpha } - 1 = C\frac{{{\mathrm{Red}}}}{{{\mathrm{Green}}}} - 1.$$

Therefore,

1 $$\frac{{[{\mathrm{AIMP}}2 - {\mathrm{DX}}2]}}{{[{\mathrm{AIMP}}2]}} \propto \frac{{{\mathrm{Red}}}}{{{\mathrm{Green}}}}.$$

For the single-cell analysis, the blue channel was used for single-cell detection. Connected pixels of the blue channel were identified using an area threshold and labeled as a nucleus. Red or green signals were assigned to the nearest nucleus based on the distance to the center of each nucleus. The red-to-green ratio was calculated by dividing the pixel area from the red channel to that of the green channel for every cell.

### Transfection

siRNAs were transfected to adherent cells using Lipofectamine 3000 (Life Technologies, MA, USA) following the manufacturer’s protocol. For suspension cells, the Neon electroporation system (1350 V, 35 ms, once for HL-60; 1350 V, 20 ms, twice for Namalwa; 1050 V, 30 ms, once for KMS-12-BM; 1100 V, 20 ms, once for ML-1) was used. siRNAs were purchased from Bioneer Korea Inc. or Genolution, and their sequences are provided in Supplementary Table 5.

### qPCR for the gene expression analysis

For qPCR, purified RNA was treated with DNase I (Takara) and reverse-transcribed using RevertAid reverse transcriptase (Thermo Fisher Scientific) with random hexamer primers. Synthesized cDNA was amplified by SensiFAST SYBR Lo-Rox Kit (Bioline) and analyzed. Primers used in this study are provided in Supplementary Table 3.

### Western blotting

Total cell lysates were prepared by sonication in the lysis buffer (50 mM Tris-HCl pH 8.0, 100 mM KCl, 0.5% NP-40, 10% glycerol, and 1 mM DTT) supplemented with protease inhibitor cocktail III (Calbiochem) and phosphatase inhibitor cocktail I (AG Scientific). A total of 30–50 μg of protein sample was separated on an SDS–PAGE gel and transferred to a PVDF membrane using the Amersham semidry transfer system. Primary antibodies used in this study are: AIMP2 (Biocon, BC-02-14), AIMP2-DX2 (Creative Biolabs, HPAB-M0004-YC), GAPDH (Santa Cruz Biotechnology, sc-32233), and following antibodies were purchased from Cell Signaling Technology: p-p53 (9284 T), PUMA (12450 T), and TUBB (86298 S). All antibodies were used with 1:1000 dilution except for AIMP2 and AIMP2-DX2, which were used with 1:500 dilution and 1:15000 dilution, respectively.

### Cell viability

MTT assay was performed to measure the cell viability. A total of 100 µL of cell suspension was transferred into a 96-well plate. A total of 20 µL of MTT solution (5 mg/mL in PBS, filtered) was added, and cells were incubated for 3.5 h at 37 °C in a humidified 5% CO_2_ incubator. Cells were then pelleted by centrifugation at 1500 × *g* for 5 min, and the supernatant was carefully removed. Cells were resuspended in 100 µL of dimethylsulfoxide. The absorbance was measured at 545 nm using a Varioskan Lux microplate reader (Thermo Fisher Scientific).

### Reporting summary

Further information on research design is available in the Nature Research Reporting Summary linked to this article.

## Supplementary information

Supplementary information

Peer Review file

Description of additional supplementary files

Supplementary data 1

Supplementary data 2

Supplementary data 3

Supplementary data 4

Reporting summary

## Data Availability

Targeted RNA-sequencing dataset of this study has been deposited in Sequence Read Archive (SRA) with the primary accession codes PRJNA589502. The ICGC/TCGA patient RNA-sequencing data are downloaded from the NIH GDC Data Portal (https://portal.gdc.cancer.gov/) with sample ID provided in the first column of Supplementary data 1. Details regarding the ICGC/TCGA data are available on request to the corresponding author Youngil Koh. Uncropped blot and gel images from the main and supplementary figures can be found in Supplementary Fig. 7. All other data needed to evaluate the conclusions in the paper are present in the paper and/or supplementary information. Source data can be found in Supplementary data 1–4.

## Code availability

The MATLAB scripts used for the RNA-smFISH image analyses are available upon request to the corresponding authors.
