# Supplementary material for: Single-cell analysis of AIMP2 splice variants informs on drug sensitivity and prognosis in hematologic cancer
Source: Commun Biol. 2020 Oct 30;3:630. doi: 10.1038/s42003-020-01353-x (PMC7599330; doi:10.1038/s42003-020-01353-x)
Supplement: Supplementary file 1 — Supplementary information [file 42003_2020_1353_MOESM1_ESM.pdf]

Supplementary figures

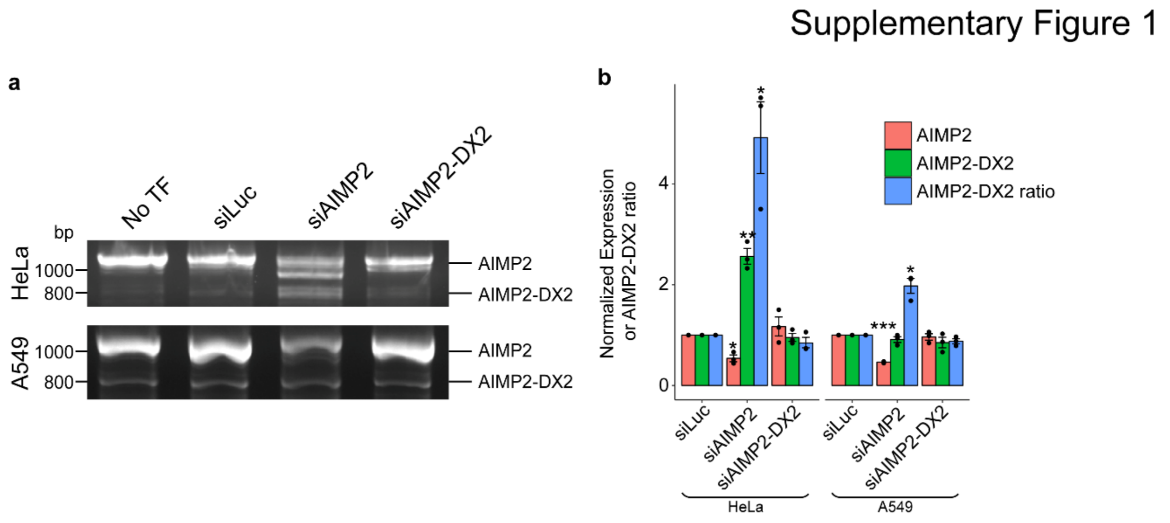

Supplementary Fig. 1. RNA-smFISH method validation. a PCR-amplified bands for AIMP2 and AIMP2-DX2 mRNA from HeLa (top) or A549 (bottom) cells. The lower band indicates AIMP2-DX2. **b** Quantified PCR band intensities in the siRNA transfected cells. Quantitation was normalized to that of the siLuc transfected cells. The average of three biological replicates are plotted with error bars indicate s.e.m. (*) $P < 0.05$, (**) $P < 0.01$, (***) $P < 0.001$. For statistical analysis, a one-tailed t-test with unequal variance was used.

Supplementary Figure 2

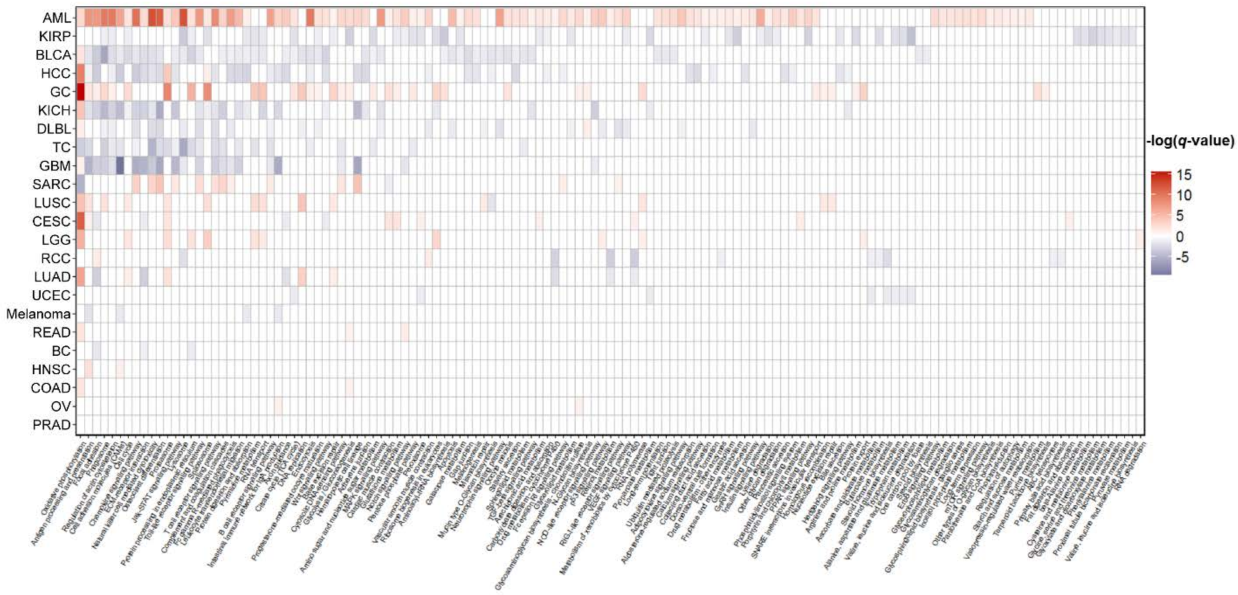

Supplementary Fig. 2. Differentially expressed gene set analysis of the ICGC/TCGA database. Differentially expressed pathways according to the AIMP2-DX2/AIMP2 expression ratio across all cancer types in the ICGC/TCGA database. The generally applicable gene-set enrichment method was applied to identify significantly dysregulated gene sets by the AIMP2-DX2/AIMP2 expression ratio. Each tile in this figure denotes $-\log_{10}$(q-values) of a specific pathway in each cancer type. A positive correlation is indicated by red while a negative correlation is indicated by color.

Abbreviations: AML, acute myeloid leukemia; BLCA, bladder urothelial carcinoma; KICH, kidney chromophobe; DLBL, diffuse large B-cell lymphoma; KIRP, kidney renal papillary cell carcinoma; HCC, hepatocellular carcinoma; LGG, low-grade glioma; GBM, glioblastoma multiforme; LUAD, lung adenocarcinoma; LUSC, lung squamous cell carcinoma; TC, thyroid

carcinoma; GC, gastric adenocarcinoma; CESC, cervical squamous cell carcinoma and endocervical adenocarcinoma; SARC, sarcoma; OV, ovarian serous cystadenocarcinoma; BC, breast invasive carcinoma; COAD, colon adenocarcinoma; READ, rectum adenocarcinoma; RCC, kidney renal clear cell carcinoma; PRAD, prostate adenocarcinoma; HNSC, head and neck squamous cell carcinoma; UCEC, uterine corpus endometrial carcinoma;

Supplementary Figure 3

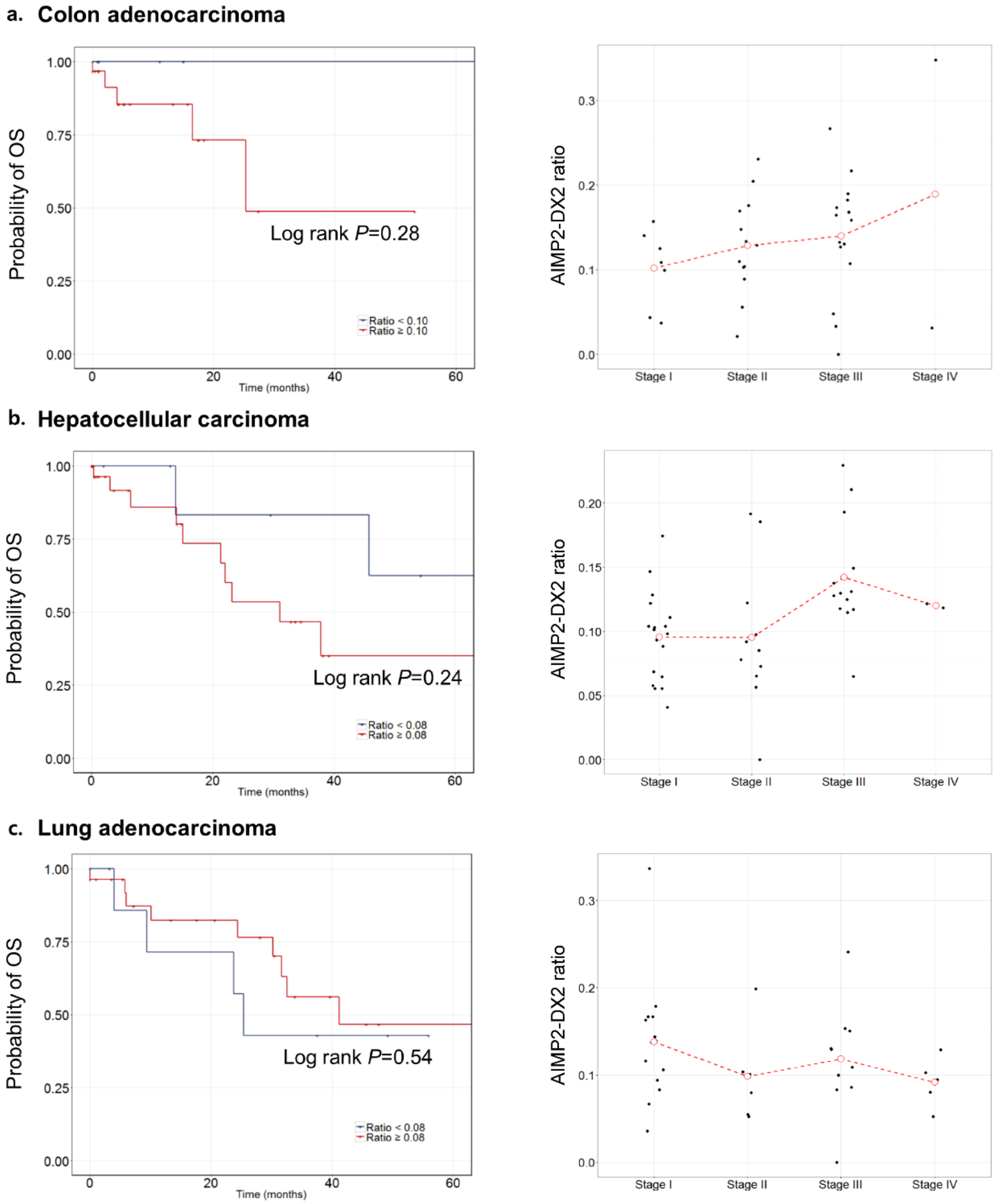

Supplementary Fig. 3. The implication of the AIMP2-DX2/AIMP2 ratio for OS and TNM stage for three cancer types in the ICGC/TCGA database: a colon carcinoma, **b** hepatocellular carcinoma, and **c** lung adenocarcinoma. For each cancer type, a Kaplan–Meier curve for OS and a

scatter plot demonstrating the distribution of AIMP2-DX2/AIMP2 expression ratio over the TNM stage are illustrated. The cutoff value for stratifying two groups in Kaplan–Meier curves was set to the first quartile value of the AIMP2-DX2/AIMP2 expression ratio. A solid red line indicates AML samples with an AIMP2-DX2/AIMP2 expression ratio ≥ cutoff, while a solid blue line corresponds to those with an AIMP2-DX2/AIMP2 expression ratio < cutoff.

Abbreviations: OS, Overall survival.

Supplementary Figure 4

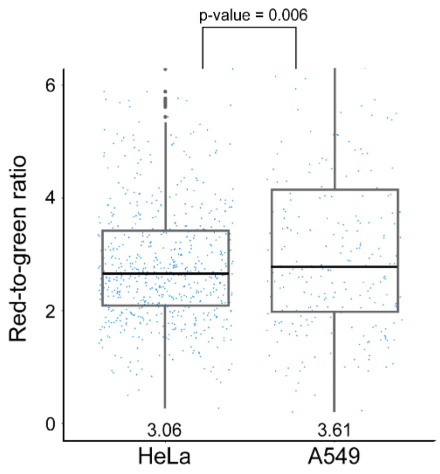

Supplementary Fig. 4. The single-cell analysis for adherent cells. Box plot of the RNA-smFISH red-to-green signal ratio of the single-cell analysis for the adherent cell lines. Numbers on the bottom of the box indicate the average rattio. n = 653 for HeLa and n = 260 for A549 cells. Box plot features: center line, median; box limits, upper and lower quartiles; whiskers, 1.5x interquartile range; points, individual red-to-green ratio. For statistical analysis, a one-tailed t-test with unequal variance was used.

Supplementary Figure 5

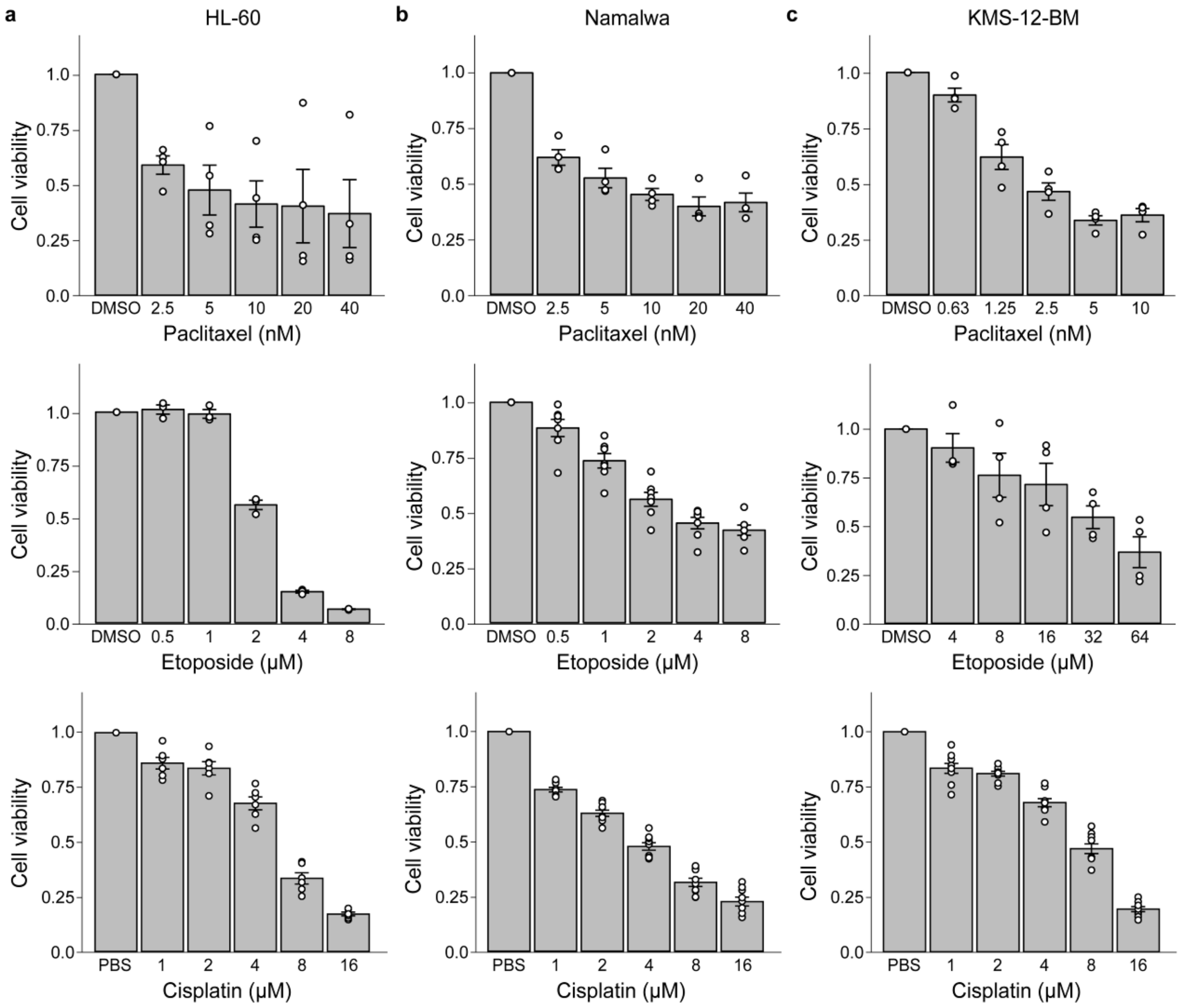

Supplementary Fig. 5. The effect of anti-cancer drug treatment on cell viability of hematologic cancers. a-c Treatment with paclitaxel, etoposide, or cisplatin results in decreased cell viability in HL-60 cells **a**, Namalwa cells **b**, and KMS-12-BM cells **c**. n = 4 for paclitaxel in all three cell lines. n = 3 for HL-60, n = 7 for Namalwa, n = 4 for KMS-12-BM for etoposide. n = 6 for HL-60, n = 9 for Namalwa, n = 9 for KMS-12-BM for cisplatin. For all data presented, the average is shown with error bars indicate s.e.m.

Supplementary Figure 6

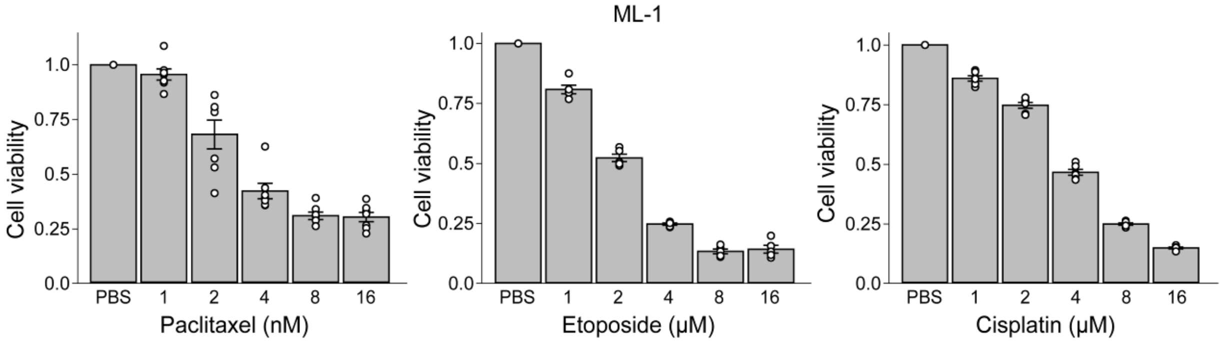

Supplementary Fig. 6. The effect of anti-cancer drug treatment on cell viability of ML-1 cells. Treatment of paclitaxel, etoposide, or cisplatin results in decreased cell viability in ML-1 cells. n = 7 for paclitaxel, n = 5 for etoposide, n = 6 for cisplatin. For all data presented, the average is shown with error bars indicate s.e.m.

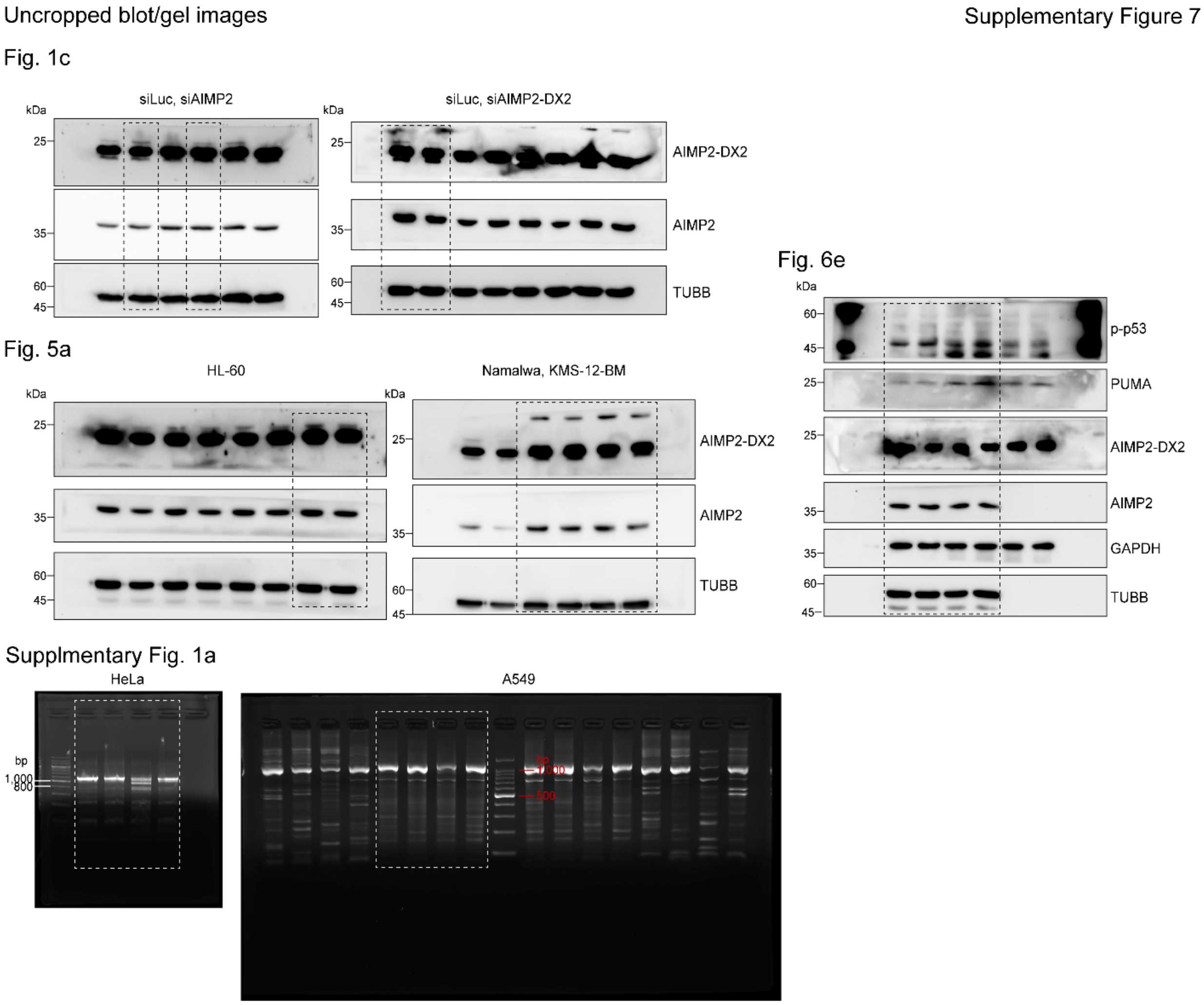

Supplementary Fig. 7. Uncropped blot/gel images from main and supplementary figures.

Supplementary Table 1. Clinical characteristics of 753 samples from the ICGC/TCGA database. Only samples with whole transcriptome data were included in this study.

Characteristics	Value
Age, mean (range)	59 (17-90)
Gender, n (%)	
Male	347 (46.1)
Female	406 (53.9)
Disease, n (%)	
Bladder urothelial carcinoma	22 (2.9)
Acute myeloid leukemia	19 (2.5)
Glioblastoma multiforme	29 (3.9)
Lung adenocarcinoma	37 (4.9)
Ovarian serous cystadenocarcinoma	20 (2.7)
Lung squamous cell carcinoma	41 (5.4)
Liver hepatocellular carcinoma	49 (6.5)
Breast invasive carcinoma	84 (11.2)
Thyroid carcinoma	44 (5.8)
Stomach adenocarcinoma	25 (3.3)
Colon adenocarcinoma	43 (5.7)
Skin cutaneous melanoma	37 (4.9)
Rectum adenocarcinoma	14 (1.9)
Kidney renal clear cell carcinoma	37 (4.9)
Prostate adenocarcinoma	18 (2.4)
Cervical squamous cell carcinoma	18 (2.4)
Diffuse large B-cell lymphoma	6 (0.8)
Head and neck squamous cell carcinoma	40 (5.3)
Kidney chromophobe	46 (6.1)
Kidney renal papillary cell carcinoma	29 (3.9)
Brain lower grade glioma	17 (2.3)
Sarcoma	33 (4.4)
Uterine corpus endometrial carcinoma	45 (6.0)

Abbreviations: ICGC, the International Cancer Consortium; TCGA, The Cancer Genome Atlas.

Supplementary Table 2. Targeted RNA-sequencing of 16 cancer cell lines and the results of RNA-smFISH analysis

Cell line	AIMP2 (read count)	AIMP2-DX2 (read count)	AIMP2-DX2 expression ratio	smFISH (red-to-green ratio)
HL-60	6424	679	10.57%	6.64
ML-1	6263	525	8.38%	8.43
SK-LU-1	10316	745	7.22%	
536MM	8291	594	7.16%	5.03
CEM	12899	786	6.09%	
NCI-H460	21081	1260	5.98%	
HS-Sultan	7239	371	5.13%	2.12
NCI-H358	25930	1167	4.50%	
SK-MES-1	41052	1593	3.88%	
A549	35737	1201	3.36%	3.20
NCI-H1299	33675	1056	3.14%	
KMS-12-BM	3337	90	2.70%	1.81
EJM	6885	161	2.34%	1.21
Calu-3	59773	1173	1.96%	
HCC-1588	29954	586	1.96%	
Namalwa	4957	69	1.39%	1.39

Abbreviations: smFISH, single-molecule fluorescence ISH.

Supplementary Table 3. Primer sequences for RT-PCR and qPCR.

RT-PCR primer sequences

Gene	Forward (5′ – 3′)	Reverse (5′ – 3′)
Full-length AIMP2	TGG CCA CGT GCA GGA AG	CTG AAG TGC TCA CAG AGG GTC
AIMP2-DX2	TGG CCA CGT GCA GGA TT	CTG AAG TGC TCA CAG AGG GTC
AIMP2	GTC GTG ACC TCT GAC GGT TT	TGA CAC TTG ACA ACT GGC CTA

qPCR primer sequences

Gene	Forward (5′ – 3′)	Reverse (5′ – 3′)
AIMP2	GCC GAT GTA CCA GGT AAA GC	ATC ATC TTG GCG GGA CTC AA
PUMA	CGA CCT CAA CGC ACA GTA CGA	AGG CAC CTA ATT GGG CTC CAT
BAX	TGG AGC TGC AGA GGA TGA TTG	GAA GTT GCC GTC AGA AAA CAT G
p21	AGG TGG ACC TGG AGA CTC TCA G	TCC TCT TGG AGA AGA TCA GCC G
p53	ACC TAT GGA AAC TAC TTC CTG AAA	CTG GCA TTC TGG GAG CTT CA
p38	CCC CGC TTA TCT CAT TAA CAG G	TCT TCT CCA GCA AGT CGA CAG
JNK	AGG ACT GCA GGA ACG AGT TT	CTC CCA TGA TGC ACC CAA CT
ERK1	CCT GCG ACC TTA AGA TTT GTG ATT	CAG GGA AGA TGG GCC GGT TAG AGA
ERK2	GCG CGG GCC CGG AGA TGG TC	TGA AGC GCA GTA AGA TTT TT
ERK5	CCT CTG TAG CGG CCA AGA AC	GGA GGA CAC CAC TCC ATA GG
MEK1	CTC TGC AGT TAA CGG GAC CA	CTC CCA CCT TCT GCT TCT GG
MEK2	TGA ACG AGC CAC CTC CTA AG	AGG TGT GGT TTG TGA GCA TCT
JAK1	CAT CCC CAT TAC GGT GCT GT	CAG ATT TCC CAG AGC GTG GT
JAK2	TGG AGG GAA CAT CCA CCT CT	TCT GCC TCA GAT TTC CCA AGG
JAK3	AGC TCT ATG CCT GCC AAG AC	GGC ACC TGT ATT GTC GCC TA
STAT1	TGA TGG CCC TAA AGG AAC TGG	AGA GCC CAC TAT CCG AGA CA
STAT2	CCG GGA CAT TCA GCC CTT TT	GTT CCA ATT GGG CCC TCT GA
STAT3	GAC ATT CCC AAG GAG GAG GC	TAT TGC TGC AGG TCG TTG GT
STAT4	ATC GTA CGT TGG TCG TGG TC	TTC CTT GCA GAA CTT GGC CC
STAT5A	TAT GTG TTT CCT GAC CGC CC	ACT CAG GGA CCA CTT GCT TG
STAT5B	ACA TTA AGG CCA CCC AGC TC	GCC CCA GCT TGA TCT TCA GT
STAT6	GGC ATC TTC TGG GTG ACT GG	GGT GCT GGA CAG TGT CTG AA
GAPDH	CTC CTC CAC CTT TGA CGC TG	TCC TCT TGT GCT CTT GCT GG

Supplementary Table 4. Information on the 17 cell lines used in this study

#	Cell line	Disease	Culture condition	Resource	cat. No.
1	HL-60	Acute myeloid leukemia	90% RPMI 1640 + 10% h.i. FBS	DSMZ	ACC-3
2	CEM	Acute lymphoblastic leukemia	90% RPMI 1640 + 10% h.i. FBS	DSMZ	ACC-240
3	HS-SULTAN	Burkitt's lymphoma	90% RPMI 1640 + 10% h.i. FBS	ATCC	CRL-1484
4	Namalwa	Burkitt's lymphoma	90% RPMI 1640 + 10% h.i. FBS	DSMZ	ACC-240
5	SNU-536MM	Multiple myeloma	90% RPMI 1640 + 10% h.i. FBS	SNU	
6	KMS-12-BM	Multiple myeloma	80% RPMI 1640 + 20% h.i. FBS	DSMZ	ACC-551
7	EJM	Multiple myeloma	90% RPMI 1640 + 10% h.i. FBS	DSMZ	ACC-560
8	SK-LU-1	Lung adenocarcinoma	90% MEM + 10% h.i. FBS	ATCC	HTB-57
9	A549	Lung adenocarcinoma	90% Dulbecco's MEM + 10% h.i. FBS	DSMZ	ACC-107
10	NCI-H460	Large cell lung cancer	90% RPMI 1640 + 10% h.i. FBS	DSMZ	ACC-737
11	NCI-H358	Non-small cell lung carcinoma	90% RPMI 1640 + 10% h.i. FBS	ATCC	CRL-5807
12	NCI-H1299	Non-small cell lung carcinoma	90% RPMI 1640 + 10% h.i. FBS	ATCC	CRL-5803
13	HCC-1588	Lung squamous cell carcinoma	90% MEM (with Earle's salts) + 10% h.i. FBS + 2 mM L-glutamine + 25 mM $NaHCO_3$	KCLB	71588
14	SK-MES-1	Lung squamous cell carcinoma	90% Dulbecco's MEM + 10% h.i. FBS	DSMZ	ACC-353
15	ML-1	Acute myeloid leukemia	90% MEM (with Earle's salts) + 10% h.i. FBS + 2 mM L-glutamine + 1 mM sodium pyruvate	DSMZ	ACC-464
16	Calu-3	Lung adenocarcinoma	90% MEM (with Earle's salts) + 10% h.i. FBS	DSMZ	ACC-734
17	HeLa	Cervical adenocarcinoma	90% Dulbecco's MEM + 10% h.i. FBS	KCLB	10002

Abbreviations: h.i. FBS, heat inactivated fetal bovine serum; DSMZ, Deutsche Sammlung von Mikroorganismen und Zellkulturen; ATCC, American Type Culture Collection; SNU, Seoul National University Hospital; KCLB, Korean Cell Line Bank.

Supplementary Table 5. Information on the siRNA sequences

Target	Sense (5′ – 3′)	Antisense (5′ – 3′)
siAIMP2	AGU CUA ACC UGU CUC UGC AAG CUC U	A GAG CUU GCA GAG ACA GGU UAG ACU
siAIMP2-DX2<br>(red = exon 1, black = exon 3)	CAC GUG CAG GAU UAC GGG GCG CUG A	U CAG CGC CCC GUA AUC CUG CAC GUG
siLuc	CUU ACG CUG AGU ACU UCG A	UCG AAG UAC UCA GCG UAA G
