# Supplementary material for: Single-cell analysis of AIMP2 splice variants informs on drug sensitivity and prognosis in hematologic cancer
Source: Commun Biol. 2020 Oct 30;3:630. doi: 10.1038/s42003-020-01353-x (PMC7599330; doi:10.1038/s42003-020-01353-x)
Supplement: Supplementary file 3 — Description of additional supplementary files [file 42003_2020_1353_MOESM3_ESM.docx]

**Description of Additional Supplementary Files**

Supplementary Data 1: Source data of the ICGC/TCGA database analysis used in Fig. 2b, 2c, 3a, supplementary fig 2, and supplementary fig 3.

Supplementary Data 2: Submitter Bundle IDs of AML patients presented in Fig. 3b.

Supplementary Data 3: Source data of the AML cohort analysis presented in Fig. 3c, d.

Supplementary Data 4: Source data of all cell line experiments.
