# Supplementary material for: Single-cell analysis of AIMP2 splice variants informs on drug sensitivity and prognosis in hematologic cancer
Source: Commun Biol. 2020 Oct 30;3:630. doi: 10.1038/s42003-020-01353-x (PMC7599330; doi:10.1038/s42003-020-01353-x)
Supplement: Supplementary file 8 — Reporting summary [file 42003_2020_1353_MOESM8_ESM.pdf]

nature research

Corresponding author(s): Yoosik Kim and Youngil Koh

Last updated by author(s): Sep 10, 2020

Reporting Summary

Nature Research wishes to improve the reproducibility of the work that we publish. This form provides structure for consistency and transparency in reporting. For further information on Nature Research policies, see our Editorial Policies and the Editorial Policy Checklist.

Statistics

For all statistical analyses, confirm that the following items are present in the figure legend, table legend, main text, or Methods section.

n/a	Confirmed	
☐	☒	The exact sample size (n) for each experimental group/condition, given as a discrete number and unit of measurement
☐	☒	A statement on whether measurements were taken from distinct samples or whether the same sample was measured repeatedly
☐	☒	The statistical test(s) used AND whether they are one- or two-sided<br>*Only common tests should be described solely by name; describe more complex techniques in the Methods section.*
☒	☐	A description of all covariates tested
☒	☐	A description of any assumptions or corrections, such as tests of normality and adjustment for multiple comparisons
☐	☒	A full description of the statistical parameters including central tendency (e.g. means) or other basic estimates (e.g. regression coefficient) AND variation (e.g. standard deviation) or associated estimates of uncertainty (e.g. confidence intervals)
☐	☒	For null hypothesis testing, the test statistic (e.g. F, t, r) with confidence intervals, effect sizes, degrees of freedom and P value noted<br>*Give P values as exact values whenever suitable.*
☒	☐	For Bayesian analysis, information on the choice of priors and Markov chain Monte Carlo settings
☒	☐	For hierarchical and complex designs, identification of the appropriate level for tests and full reporting of outcomes
☒	☐	Estimates of effect sizes (e.g. Cohen's d, Pearson's r), indicating how they were calculated

Our web collection on statistics for biologists contains articles on many of the points above.

Software and code

Policy information about availability of computer code

Data collection	Targeted RNA-sequencing data of the hematologic malignant cell lines used in our study were generated and processed by using Ion Torrent Suite Software v4.0.2. Patient RNA expression data from ICGC/TCGA database were downloaded from the Cancer Genomics Hub (CGHub) of the sequencing programs of the National Cancer Institute (NCI) using GeneTorrent v3.8.7.
Data analysis	The Binary version of the Sequence Alignment/Map (BAM) files were extracted using Samtools v1.2 (http://www.htslib.org/) from our targeted RNA-sequencing data. We used common Linux (https://www.linux.org/) command and Samtools for extracting chromosomal loci of AIMP2 gene from the original Sequence Alignment/Map (SAM) file.<br>Downloaded ICGC/TCGA patient data was mapped to human exons by Tophat2 (https://ccb.jhu.edu/software/tophat/index.shtml), and aligned data analyzed by HTSeq-count (https://htseq.readthedocs.io/en/master/) and R version 3.3.1 (http://www.r-project.org). Differentially expressed genes in RNA sequencing data were analyzed by R package, DESeq2 (https://bioconductor.org/packages/release/bioc/html/DESeq2.html), and then utilized data to identify differentially regulated pathways using another R package, Generally Applicable Gene-set Enrichment (GAGE, https://bioconductor.org/packages/release/bioc/html/gage.html).<br>For the cell line analysis, Microsoft Excel 2016 was used. For RNA-smFISH analysis, MATLAB v.2014b was used.

For manuscripts utilizing custom algorithms or software that are central to the research but not yet described in published literature, software must be made available to editors and reviewers. We strongly encourage code deposition in a community repository (e.g. GitHub). See the Nature Research guidelines for submitting code & software for further information.

Data

Policy information about availability of data

All manuscripts must include a data availability statement. This statement should provide the following information, where applicable:

- Accession codes, unique identifiers, or web links for publicly available datasets
- A list of figures that have associated raw data
- A description of any restrictions on data availability

Targeted RNA-sequencing dataset that support the findings of this study have been deposited in Sequence Read Archive (SRA) with the primary accession codes PRJNA589502 (https://www.ncbi.nlm.nih.gov/bioproject/PRJNA589502). The ICGC/TCGA patient RNA sequencing data are available on request to the corresponding author Youngil Koh. All other data needed to evaluate the conclusions in the paper are present in the paper and/or supplementary information. The main scripts used for the presented analyses are available upon request to the corresponding authors.

Field-specific reporting

Please select the one below that is the best fit for your research. If you are not sure, read the appropriate sections before making your selection.

☒ Life sciences ☐ Behavioural & social sciences ☐ Ecological, evolutionary & environmental sciences

For a reference copy of the document with all sections, see nature.com/documents/nr-reporting-summary-flat.pdf

Life sciences study design

All studies must disclose on these points even when the disclosure is negative.

Sample size	Due to retrospective validation design, sample size calculation was not performed before study. We utilized all AML samples available for AIMP2-DX2 analysis at SNUH. For cell study, we used at least 3 biological replicates to test the statistical significance of our data.
Data exclusions	No data were excluded from the analysis.
Replication	We performed at least 3 biological replicates to show the reproducibility of our data.
Randomization	Not applicable.
Blinding	Not applicable.

Reporting for specific materials, systems and methods

We require information from authors about some types of materials, experimental systems and methods used in many studies. Here, indicate whether each material, system or method listed is relevant to your study. If you are not sure if a list item applies to your research, read the appropriate section before selecting a response.

Materials & experimental systems

n/a	Involved in the study	
☐	☒	Antibodies
☐	☒	Eukaryotic cell lines
☒	☐	Palaeontology and archaeology
☒	☐	Animals and other organisms
☐	☒	Human research participants
☒	☐	Clinical data
☒	☐	Dual use research of concern

Methods

n/a	Involved in the study	
☒	☐	ChIP-seq
☒	☐	Flow cytometry
☒	☐	MRI-based neuroimaging

Antibodies

Antibodies used	Mouse monoclonal anti-AIMP2, Biocon, Cat#BC-02-14<br>Rabbit monoclonal anti-AIMP2-DX2 (H5), Creative Biolabs, HPAB-M0004-YC<br>Mouse monoclonal anti-GAPDH (6C5), Santa Cruz Biotechnology, Cat#sc-32233<br>Rabbit polyclonal anti-p-p53 (Ser15), Cell Signaling Technology, Cat#9284T<br>Rabbit monoclonal anti-PUMA (D30C10), Cell Signaling Technology, Cat#12450T<br>Mouse monoclonal anti-TUBB (D3U1W), Cell Signaling Technology, Cat#86298S
Validation	The validation of commercial antibodies used in this study were posted in manufacturer's website.

Eukaryotic cell lines

Policy information about cell lines

Cell line source(s)	HL-60 (DSMZ), CEM (DSMZ), HS-SULTAN (ATCC), Namalwa (DSMZ), SNU-536MM (SNU), KMS-12-BM (DSMZ), EJM (DSMZ), SK-LU-1 (ATCC), A549 (DSMZ), NCI-H460 (DSMZ), NCI-H358 (ATCC), NCI-H1299 (ATCC), HCC-1588 (KCLB), SK_MES-1 (DSMZ), ML-1 (DSMZ), Calu-3 (DSMZ), HeLa (KCLB).
Authentication	The cell line SNU-536MM was internally established at Seoul National University Hospital using a bone marrow sample of multiple myeloma patients.<br>Other all cell lines were authenticated by STR profiling.
Mycoplasma contamination	All cell lines were tested regularly for mycoplasma contamination.
Commonly misidentified lines<br>(See ICLAC register)	According to the ICLAC register, cross-contamination has been reported in HS-Sultan cells where no authentic stock is known. We used HS-Sultan cell line authenticated by STR profiling analysis.

Human research participants

Policy information about studies involving human research participants

Population characteristics	For a total of 51 AML patients included in this analysis, the median age was 54.3 years (range 20.4–83.8 years; Table 1), 23 out of 51 patients (45.1%) were female. The most common French–American–British (FAB) classification subtype was M2 (33.3%); most of the patients (64.7%) were classified into an intermediate-risk group by the Medical Research Council (MRC) criteria.
Recruitment	AML patients who visited SNUH for diagnosis and treatment were recruited consecutively following informed consent.
Ethics oversight	This protocol was approved by the Seoul National University Hospital Institutional Review Board (IRB approval number: 1201-099-396). This study was conducted in accordance with the Declaration of Helsinki provisions.

Note that full information on the approval of the study protocol must also be provided in the manuscript.
