# Supplementary material for: Single-cell analysis of AIMP2 splice variants informs on drug sensitivity and prognosis in hematologic cancer
Source: Commun Biol. 2020 Oct 30;3:630. doi: 10.1038/s42003-020-01353-x (PMC7599330; doi:10.1038/s42003-020-01353-x)
Supplement: Supplementary file 2 — Peer Review file [file 42003_2020_1353_MOESM2_ESM.pdf]

Reviewers' comments:

Reviewer #1 (Remarks to the Author):

Comments to the Author
This study aimed to better understand the functions of AIMP2-DX2 and its clinical implications in hematologic cancer. Overviewing and emphasizing the effects of cells to anti-cancer drugs by down-regulating AIMP2-DX2 suggests its clinical implications in hematologic cancer. Towards that goal, the authors suggested AIMP2-DX2 as a potential biomarker and a therapeutic target for hematologic cancer.It is also meaningful to quantify the mRNA ratio of AIMP2-DX2/AIMP2 at the single cell level. Overall, the data was close to support the conclusion. I recommended some major revisions as described below.

1.In abstract, the author pointed out that they found AIMP2-DX2/AIMP2 expression ratio was strongly correlated with the poor prognosis of major cancer. But survival analysis was performed only in AML, neither PS nor PFS curves were studied in other tumors, the conclusions needed more evidences.
2. In figure 3a,19 AML samples from ICGC/TCGA databases were analyzed and P value of overall survival was 0.16, which means no significant difference. Please provide median survival time to further demonstrate the prognostic significance of this figure.
3. The major signaling pathways were only analyzed in the database, more data should be presented to verify its functions in cell lines.
4. It's worth noting that 95% of acute promyelocytic leukemia can be cured, considering ATRA and ATO have been proved very effective in the treatment of APL, the significance of drug sensitivity of HL-60 for paclitaxel and etoposide might be limited. More details might be needed to clarify the practical significance of these two drugs.
5. line 276 to 278, the conclusion targeting the adjuvant effects on anti-cancer drugs, authors should give more experimental evidence or explanations to prove it.

Reviewer #2 (Remarks to the Author):

The study by Ku et al compares various techniques to determine the mRNA expression ratios of the non-enzymatic tRNA synthase complex subunit AIMP2 with is splice variant AIMP2-DX2. Specifically, they have designed and validated smRNA-FISH probes that label both transcripts within the same cell. Another part of the study deals with correlation analyses of AIMP2-DX2 expression (or the ratio between the splice variant and the full length mRNA) using either publically available data or mRNA measurements from an AML cohort of 51 patients. Altogether, these results suggest (but by no means proof) that higher AIMP2-DX2 expression might contribute to decreased overall or progression free survival. Finally, the authors try to substantiate this conclusion in paclitaxel or etoposide-treated cancer cell line experiments using transient knockdowns of AIMP2-DX2. However, again the effects are mixed. The manuscript is well written, the presentation of the data is convincing and the combination of in silico and experimental analyses is of interest for both, basic scientists and clinicians working in the cancer field. However, I suggest toning down the statements on the significance of altered AIMP2-DX2/AIMP2 levels for predicting survival rates or the response to therapy in cancer patients.
Specific points:

Fig. 1a/b: The graphical presentation of FISH probes suggests that the full length mRNA will bind both probes and thus should reveal some degree of merged colors in all spots. However, in the original images, there are only few merged signals. Along this line: how is the existence of purely green fluorescence signals explained? Theoretically, such a spot should consist of exon 2 only? The authors need to provide more explanation for this phenomenon. They should also show (and quantify) negative controls, in which all detection reagents have been added except the primary probes.

Fig. 2b: the abbreviations of all cancer types need to explained in the legend.

Fig. 3a: The ratio values of 0.05 suggest that there is a very low expression of AIMP2-DX2 compared to the full length transcript. Considering this and without any data at the protein level, how can the author conclude that there is any functional relevance for the splice variant?

Fig.4a/b: the median survival rates and their variations should be indicated.

Fig. 5d: What is the difference between RNA FISH (white bars) and single cell (gray bars)?

Fig.6: It is unclear from the presentation and also from the legends / methods if the left graphs are derived from independent dose response curves compared to the right graphs which show the effects of knockdowns. Ideally, the reduction of AIMP2-DX2 should be shown alongside each of the different experiments to validate that there was comparable reduction of the mRNA (or even better of the protein) across all conditions.

Page 10, lines 207-208: This statement on the strong evidence for the carcinogenic role AIMP2-DX is not justified based on the results shown in this study. If at all, the data reveal some interesting but variable correlations.

Page 13, lanes 276-278: Similar, none of the data show strong enough inhibitory effects in vitro to suggest that targeting AIMP2-DX can be used as an adjuvant in p53-dependent tumors in a clinical setting.

Page 23: “..database.21 (To) A..”- There is a typo.

Response to Reviewers

We would like to thank the reviewers for the constructive comments and helpful suggestions. We hope you will find our point-by-point response clear.

Reviewer #1:

This study aimed to better understand the functions of AIMP2-DX2 and its clinical implications in hematologic cancer. Overviewing and emphasizing the effects of cells to anti-cancer drugs by down-regulating AIMP2-DX2 suggests its clinical implications in hematologic cancer. Towards that goal, the authors suggested AIMP2-DX2 as a potential biomarker and a therapeutic target for hematologic cancer. It is also meaningful to quantify the mRNA ratio of AIMP2-DX2/AIMP2 at the single cell level. Overall, the data was close to support the conclusion. I recommended some major revisions as described below.

[Response] Thank you for the comment. Our point-by-point response to the suggestions is below. We revised the manuscript to reflect these changes.

1. In abstract, the author pointed out that they found AIMP2-DX2/AIMP2 expression ratio was strongly correlated with the poor prognosis of major cancer. But survival analysis was performed only in AML, neither PS nor PFS curves were studied in other tumors, the conclusions needed more evidences.

[Response] In the revised manuscript, we also analyzed the prognostic values, such as OS and TNM, of *AIMP2-DX2/AIMP2* expression ratio in other types of cancer. We found that for colon carcinoma and hepatocellular carcinoma, patients with an *AIMP2-DX2/AIMP2* expression ratio ≥ Q_1 tend to exhibit an inferior OS. Although statistically insignificant, the *AIMP2-DX2/AIMP2* expression ratio showed an increasing tendency with the TNM stage. In contrast, such a prognostic value and correlation with the TNM stage were not evident in other cancer types, including lung adenocarcinoma. These additional results are provided in the Supplementary Figure 3. Comments on the relation between *AIMP2-DX2/AIMP2* expression ratio and poor prognosis in the major cancers are added on page 10 of the revised manuscript.

p. 10, line 218.

[The implication of the *AIMP2-DX2/AIMP2* expression ratio for OS and Tumor-Node-Metastasis (TNM) stage was further investigated in other cancer types (Supplementary Fig. 3). Similar to AML, samples with an *AIMP2-DX2/AIMP2* expression ratio ≥ Q_1 tended to exhibit an inferior OS in colon carcinoma (Log-rank P=0.28), and hepatocellular carcinoma (Log-rank P=0.24). For these cancer types, although statistically insignificant, the *AIMP2-DX2/AIMP2* expression ratio showed a tendency toward increasing along with the stage. Additional studies with a larger patient number will be able to delineate clinical implications of the *AIMP2-DX2/AIMP2* expression ratio in these cancers. In contrast, such a prognostic value and correlation with the

stage were not evident in other cancer types. Analysis of lung adenocarcinoma is shown as an example in Supplementary Fig. 3c.]

2. In figure 3a, 19 AML samples from ICGC/TCGA databases were analyzed and P value of overall survival was 0.16, which means no significant difference. Please provide median survival time to further demonstrate the prognostic significance of this figure.

[Response] Per reviewer's suggestion, we provided the median survival and variations of patients with the ICGC/TCGA database in the revised manuscript. Of note, while reanalyzing the data, we found one patient data missing for Fig. 3a and corrected the Kaplan-Meier curve and log-rank *P*. Additional comments on the median survival and variations of patients from the databases are added on page 9 of the revised manuscript.

p. 9, line 182.

[Using a cutoff ratio of 0.04, which was the first quartile value (Q_1) of the *AIMP2-DX2/AIMP2* expression ratio in AML, a Kaplan–Meier curve for overall survival (OS) showed that patients with an *AIMP2-DX2/AIMP2* expression ratio $\geq Q_1$ tended to exhibit poor OS (median survival 47.7 months) compared to those with an *AIMP2-DX2/AIMP2* expression ratio $< Q_1$ (median survival not reached; Log-rank P=0.25; Fig. 3a).]

3. The major signaling pathways were only analyzed in the database, more data should be presented to verify its functions in cell lines.

[Response] We thank the reviewer for the constructive comment. To address the concern, we analyzed the effect of AIMP2-DX2 knockdown on major cancer signaling pathways in ML-1 AML cells. We choose ML-1 because, according to our targeted RNA-seq (Supplementary Table 2), ML-1 cells showed a high expression ratio of *AIMP2-DX2/AIMP2.* We further confirmed this using the developed RNA-FISH image analysis (Fig. 6a).

We found that the knockdown of AIMP2-DX2 resulted in the downregulation of representative genes of the MAPK and JAK-STAT signaling pathways, which validates our analysis of the ICGC/TCGA database that *AIMP2-DX2/AIMP2* expression ratio is positively correlated with these pathways. In the case of p53 signaling, we found that its downstream signaling became strongly induced in AIMP2-DX2 deficient cells when etoposide was treated. These new results are incorporated into the revised Fig. 6 and page 14 and 15 of the manuscript.

p. 14, line 316.

[Considering the ICGC/TCGA analysis that several cancer pathways showed a positive correlation with the ratio of *AIMP2-DX2/AIMP2* in AML, we extended our analysis using ML-1 AML cells. First, we applied the RNA-smFISH approach to quantitate the *AIMP2-DX2/AIMP2* expression ratio in ML-1 cells. We found that the red-to-green ratio has a mean value of 8.43, which categorizes ML-1 to a high expression group (Fig. 6a). This is consistent with the targeted

RNA-sequencing result that ML-1 is one of the cell lines with a high expression ratio of *AIMP2-DX2/AIMP2* (Supplementary Table 2). Next, we utilized these cells to verify the result of the ICGC/TCGA database analysis that *AIMP2-DX2/AIMP2* expression ratio correlates with major cancer signaling pathways in AML. We knocked down the expression of AIMP2-DX2 by transfecting siAIMP2-DX2 and examined its effect on components of p53, MAPK, and JAK-STAT signaling pathways using RT-qPCR. Among the genes examined, *p38α* and *c-Jun N-terminal kinase 1* (*JNK1)* of MAPK signaling and *JAK1-3* and *STAT1* of JAK-STAT signaling showed a positive correlation with the *AIMP2-DX2* expression (Fig. 6b). This is consistent with our analysis of the ICGC/TCGA database that AIMP2-DX2 is positively correlated with MAPK and JAK-STAT signaling pathways in AML (Fig. 2c and Fig. 3b). On the contrary, we could not find a correlation between p53 signaling pathway and *AIMP2-DX2* expression. Nevertheless, our data suggest that AIMP2-DX2 may regulate tumorigenesis in AML.]

p. 15, line 337.

[We then examined the activation of the p53 signaling pathway when cells were treated with etoposide using qPCR and western blotting. Although our earlier analysis showed no correlation between p53 signaling and AIMP2-DX2 without any stressor, when we treated etoposide, *p21* and *PUMA* mRNAs were strongly induced in AIMP2-DX2-deficient cells (Fig. 6d). Moreover, we found increased phosphorylation of p53 and increased expression of PUMA protein when etoposide was treated in AIMP2-DX2 knockdown cells (Fig. 6e).]

4. It's worth noting that 95% of acute promyelocytic leukemia can be cured, considering ATRA and ATO have been proved very effective in the treatment of APL, the significance of drug sensitivity of HL-60 for paclitaxel and etoposide might be limited. More details might be needed to clarify the practical significance of these two drugs.

[Response] We clarify that HL-60 was chosen because it showed a high expression ratio of *AIMP2-DX2/AIMP2*. In addition, we used paclitaxel and etoposide because they require the p53 signaling system to induce apoptosis. However, we agree with the reviewer's concern that using HL-60 is misleading, and the significance of paclitaxel and etoposide on HL-60 have limited clinical implications.

To address the reviewer's comment, we analyzed an AML cell line ML-1, which showed a high expression ratio of *AIMP2-DX2/AIMP2* according to both targeted RNA-seq and RNA-smFISH analyses (Fig. 6a, b). We then examined the effect of AIMP2-DX2 knockdown on the sensitivity to paclitaxel and etoposide (Fig. 6c). Similar to the case of HL-60, knockdown of AIMP2-DX2 resulted in increased susceptibility to these anti-cancer drugs. Moreover, we also tested an additional drug, cisplatin, which is more clinically relevant anti-cancer drug for AML. We found that, when AIMP2-DX2 was knocked down, HL-60 and ML-1 cells showed increased sensitivity, KMS-12-BM showed no effect, and Namalwa showed decreased sensitivity (Fig. 5b-d). These new data are incorporated on page 14.

p. 14, line 303.

[We performed an analogous set of experiments, but this time using etoposide or cisplatin instead of paclitaxel to trigger apoptosis. We chose etoposide because it induces DNA damage and initiates p53 signaling[17,18]. In addition, cisplatin is a p53 dependent anti-cancer drug[19,20] currently being used to treat refractory lymphomas and AML[21-24]. We found that only the high *AIMP2-DX2* expressing HL-60 cells could be sensitized to etoposide and cisplatin via knockdown of AIMP2-DX2 (Fig. 5b). At the same time, transfecting Namalwa cells with siAIMP2-DX2 again showed the adverse effect where it partially rescued cell death by etoposide and cisplatin (Fig. 5c). Lastly, targeting AIMP2-DX2 did not have any effect on apoptotic response in KMS-12-BM cells (Fig. 5d). Together, our results clearly indicate that targeting AIMP2-DX2 expression can be used to enhance the effectiveness of anti-cancer drugs such as paclitaxel, etoposide, and cisplatin that rely on the p53 signaling pathway.]

5. line 276 to 278, the conclusion targeting the adjuvant effects on anti-cancer drugs, authors should give more experimental evidence or explanations to prove it.

[Response] Per the reviewer's suggestion, we revised our manuscript to make our conclusions to reflect our data clearly. We removed the phrase "the adjuvant effects on anti-cancer drugs" and specified that targeting AIMP2-DX2 expression can increase the sensitivity to anti-cancer drugs that rely on p53 signaling. Modified texts are on pages 14 and 15 of the revised manuscript.

p. 14, line 311.

[Together, our results clearly indicate that targeting AIMP2-DX2 expression can be used to enhance the effectiveness of anti-cancer drugs such as paclitaxel, etoposide, and cisplatin that rely on the p53 signaling pathway.]

p. 15, line 343.

[Collectively, these data support that the downregulation of AIMP2-DX2 in ML-1 cells can affect their response to anti-cancer drugs by modulating p53 signaling pathway.]

Reviewer #2:

The study by Ku et al compares various techniques to determine the mRNA expression ratios of the non-enzymatic tRNA synthase complex subunit AIMP2 with is splice variant AIMP2-DX2. Specifically, they have designed and validated smRNA-FISH probes that label both transcripts within the same cell. Another part of the study deals with correlation analyses of AIMP2-DX2 expression (or the ratio between the splice variant and the full-length mRNA) using either publically available data or mRNA measurements from an AML cohort of 51 patients. Altogether, these results suggest (but by no means proof) that higher AIMP2-DX2 expression might contribute to decreased overall or progression free survival. Finally, the authors try to substantiate this conclusion in paclitaxel or etoposide-treated cancer cell line experiments using transient knockdowns of AIMP2-DX2. However, again the effects are mixed. The manuscript is well written, the presentation of the data is convincing and the combination of in silico and experimental analyses is of interest for both, basic scientists and clinicians working in the cancer field. However, I suggest toning down the statements on the significance of altered AIMP2-DX2/AIMP2 levels for predicting survival rates or the response to therapy in cancer patients.

[Response] We thank the reviewer for the comments. Our point-by-point response to the suggestions is below. We have revised the manuscript to reflect these changes.

1. Fig. 1a/b: The graphical presentation of FISH probes suggests that the full length mRNA will bind both probes and thus should reveal some degree of merged colors in all spots. However, in the original images, there are only few merged signals. Along this line: how is the existence of purely green fluorescence signals explained? Theoretically, such a spot should consist of exon 2 only? The authors need to provide more explanation for this phenomenon.

[Response] We thank the reviewer for the detailed comment. The lack of merged signals is due to limitations on the number of ZZ pairs for detection. The recommended number of ZZ pairs for smFISH is about 20. However, the exon 2 of the AIMP2 gene is only 207 bp, and we could only design 5 ZZ probe pairs. To detect exons 1, 3, and 4 (C2 probes), we were able to design 16 ZZ pairs, but this was still not enough to capture all *AIMP2* and *AIMP2-DX2* mRNAs. As our probes only visualized a fraction of these mRNAs, there existed purely green and red fluorescence signals and only a few merged signals. We rigorously validated our approach by analyzing the negative control (Fig. 1b), the effect of AIMP2 and AIMP2-DX2 knockdown (Fig. 1d-i), and compared the RNA-smFISH analysis results with that of targeted RNA-seq (Fig. 4). In the revised manuscript, we incorporated a discussion regarding the number of ZZ pairs and the lack of merged fluorescent foci on page 7.

p. 7, line 147.

[Of note, due to differences in the number of ZZ pairs between the two channels, we found that the efficiencies of the probes in capturing their target mRNAs were different. This resulted in only a few overlapping foci because there are always more red foci than the green.]

2. They should also show (and quantify) negative controls, in which all detection reagents have been added except the primary probes.

[Response] Per the reviewer's suggestion, we conducted a negative control RNA-smFISH experiment in which all detection reagents were added except the C1 and C2 hybridization probes. We did not detect any fluorescent signal in the negative control sample (Fig. 1b). The description of the negative control experiment is provided on page 6 of the revised manuscript

p. 6, line 116.

[As a control, we performed RNA-smFISH without hybridization of C1 and C2 probes, which resulted in no fluorescent signal (Fig. 1b).]

3. Fig. 2b: the abbreviations of all cancer types need to explained in the legend.

[Response] We updated the abbreviations of all cancer types examined in the Figure 2 legend.

4. Fig. 3a: The ratio values of 0.05 suggest that there is a very low expression of AIMP2-DX2 compared to the full length transcript. Considering this and without any data at the protein level, how can the author conclude that there is any functional relevance for the splice variant?

[Response] We appreciate the constructive comment. The small ratio can have a significant effect because AIMP2-DX2 only needs to inhibit a fraction of AIMP2 that is dissociated from the MSC. Normally, AIMP2 exists as a part of the MSC, but in response to DNA damage, a small fraction of AIMP2 dissociates from the complex to induce apoptosis by protecting p53 from degradation. On the contrary, AIMP2-DX2 only exists as a free form and is not associated with the MSC. As our ratio compares the expression of *AIMP2-DX2* to total *AIMP2* (MSC-associated + free), small ratio of AIMP2-DX2 can effectively inhibit the action of AIMP2 on p53. We modified page 9 of the revised manuscript to clarify this point.

Additionally, our cell line experiments further confirm the functional relevance for the splice variant. We showed that the knockdown of AIMP2-DX2 in HL-60 and ML-1 (with the ratio of ~0.08~0.1) sensitized cells to anti-cancer drugs such as paclitaxel, etoposide, and cisplatin while analogous experiment in two cell lines with a ratio less than 0.04 (KMS-12-BM with the ratio of 0.027 and Namalwa with the ratio of 0.0139) did not become sensitized to the same anti-cancer drugs. Of note, while reanalyzing the data, we found that the Q1 threshold ratio is actually 0.04.

p. 9, line 186.

[Of note, AIMP2-DX2 exists as a free form and is not associated with MSC[7]. Considering that most AIMP2 protein exists in MSC[12] and only a fraction becomes dissociated to mediate stress response[4], a small amount of AIMP2-DX2 compared to total AIMP2 (free + MSC associated) can still act as a potent inhibitor of AIMP2.]

5. Fig.4a/b: the median survival rates and their variations should be indicated.

[Response] Per the reviewer's suggestion, we provided the median survival and variations of analyzed patient data. The description on the median survival rates and their variations are provided on page 10 of the revised manuscript.

p. 10, line 210.

[The OS of the *AIMP2-DX2* positive group (median survival 11.7 [5.03-29.6] months) was significantly inferior to that of the *AIMP2-DX2* negative group (median survival not reached) with a hazard ratio (HR) of 2.47 (95% CI, 1.14–5.34; *P*=0.022).]

p. 10, line 214.

[Similarly, the *AIMP2-DX2* positive group showed a worse PFS (median survival 5.97 [3.07-13.8] months) compared to the *AIMP2-DX2* negative group (median survival 19.93 [7.10-not reached] months; HR, 2.59; 95% CI, 1.32–5.11; *P*=0.006).]

6. Fig. 5d: What is the difference between RNA FISH (white bars) and single cell (gray bars)?

[Response] In Fig. 5d (current Fig. 4d), RNA FISH (white bars) represents fluorescent intensity quantification per image while single cell (gray bars) denotes fluorescent intensity quantification per cell. Both of them are based on RNA-smFISH image analysis. To clarify the data presentation, we changed "RNA-FISH" to "Single image" and provided a better description of these two approaches on page 12 and the figure legend of the revised manuscript.

p. 12, line 261.

[A comparison of RNA-smFISH single-image quantitation, single-cell analysis, and targeted RNA-sequencing is summarized in Fig. 4d.]

7. Fig.6: It is unclear from the presentation and also from the legends / methods if the left graphs are derived from independent dose response curves compared to the right graphs which show the effects of knockdowns. Ideally, the reduction of AIMP2-DX2 should be shown alongside each of the different experiments to validate that there was comparable reduction of the mRNA (or even better of the protein) across all conditions.

[Response] Per the reviewer's suggestion, we performed western blotting experiments to confirm the reduction of AIMP2-DX2 protein under our transfection condition. We showed that the transfection of siAIMP2-DX2 could successfully reduce the expression of the target protein in HeLa (Fig. 1c), HL-60 (Fig. 5a), and ML-1 (Fig. 6e). We could not confirm the AIMP2-DX2

expression in Namalwa and KMS-12-BM due to low AIMP2-DX2 expressions in these two cell lines.

In addition, to clarify the data presented in Fig. 6 (current Fig. 5b-d), we moved the cell viability data for siLuc control to the Supplementary Figure 5 and only presented the effect of AIMP2-DX2 knockdown on cell viability in Fig 5b-d. We modified the text on page 12 and the figure legend to clearly explain our data.

p. 12, line 274.

[To target *AIMP2-DX2*, we performed electroporation with siAIMP2-DX2 to three blood cancer cell lines. HL-60 promyelocytic leukemia cells, which expressed the highest ratio of *AIMP2-DX2/AIMP2,* clearly showed decreased expression of AIMP2-DX2 (Fig. 5a). The expression of AIMP2-DX2 in the other two cell lines was too low to be detectable. However, we optimized the electroporation condition using a control target and applied the same condition to transfect siAIMP2-DX2.]

8. Page 10, lines 207-208: This statement on the strong evidence for the carcinogenic role AIMP2-DX is not justified based on the results shown in this study. If at all, the data reveal some interesting but variable correlations.

[Response] In the revised manuscript, we performed additional experiments to examine the carcinogenic role of AIMP2-DX2 on major cancer pathways in AML to support our analysis of the public databases. We analyzed the effect of AIMP2-DX2 knockdown on major components of the p53, MAPK, and JAK-STAT signaling pathways. We found that the knockdown of AIMP2-DX2 significantly decreased the expression of *p38α* and *JNK1* mRNAs (MAPK signaling pathway) as well as JAK2 and STAT1 mRNAs (JAK-STAT signaling pathways) (Fig. 6b). This is consistent with our analysis of the ICGC/TCGA databases that *AIMP2-DX2/AIMP2* expression is positively correlated with major cancer signaling pathways in AML. In the case of p53 signaling, we found that the signaling system was more strongly induced in AIMP2-DX2 deficient cells when etoposide was treated (Fig. 5d, 5e). These new data further support that AIMP2-DX2 may have carcinogenic role in AML. The description on the effect of AIMP2-DX2 knockdown on the major cancer signaling pathways is added on pages 14 and 15 of the revised manuscript.

p. 14, line 316.

[Considering the ICGC/TCGA analysis that several cancer pathways showed a positive correlation with the ratio of *AIMP2-DX2/AIMP2* in AML, we extended our analysis using ML-1 AML cells. First, we applied the RNA-smFISH approach to quantitate the *AIMP2-DX2/AIMP2* expression ratio in ML-1 cells. We found that the red-to-green ratio has a mean value of 8.43, which categorizes ML-1 to a high expression group (Fig. 6a). This is consistent with the targeted RNA-sequencing result that ML-1 is one of the cell lines with a high expression ratio of *AIMP2-DX2/AIMP2* (Supplementary Table 2). Next, we utilized these cells to verify the result of the ICGC/TCGA database analysis that *AIMP2-DX2/AIMP2* expression ratio correlates with major

cancer signaling pathways in AML. We knocked down the expression of AIMP2-DX2 by transfecting siAIMP2-DX2 and examined its effect on components of p53, MAPK, and JAK-STAT signaling pathways using RT-qPCR. Among the genes examined, *p38α* and *c-Jun N-terminal kinase 1* (*JNK1)* of MAPK signaling and *JAK1-3* and *STAT1* of JAK-STAT signaling showed a positive correlation with the *AIMP2-DX2* expression (Fig. 6b). This is consistent with our analysis of the ICGC/TCGA database that AIMP2-DX2 is positively correlated with MAPK and JAK-STAT signaling pathways in AML (Fig. 2c and Fig. 3b). On the contrary, we could not find a correlation between p53 signaling pathway and *AIMP2-DX2* expression. Nevertheless, our data suggest that AIMP2-DX2 may regulate tumorigenesis in AML.]

p. 15, line 337.

[We then examined the activation of the p53 signaling pathway when cells were treated with etoposide using qPCR and western blotting. Although our earlier analysis showed no correlation between p53 signaling and AIMP2-DX2 without any stressor, when we treated etoposide, *p21* and *PUMA* mRNAs were strongly induced in AIMP2-DX2-deficient cells (Fig. 6d). Moreover, we found increased phosphorylation of p53 and increased expression of PUMA protein when etoposide was treated in AIMP2-DX2 knockdown cells (Fig. 6e).]

9. Page 13, lanes 276-278: Similar, none of the data show strong enough inhibitory effects in vitro to suggest that targeting AIMP2-DX can be used as an adjuvant in p53-dependent tumors in a clinical setting.

[Response] Per the reviewer's suggestion, we revised our manuscript to make our conclusions reflect our data clearly. We removed the phrase "the adjuvant effects on anti-cancer drugs" and specified that targeting AIMP2-DX2 expression can sensitize cells to anti-cancer drugs that rely on p53 signaling. Modified texts are presented on pages 14 and 15 of the revised manuscript.

p. 14, line 311.

[Together, our results clearly indicate that targeting AIMP2-DX2 expression can be used to enhance the effectiveness of anti-cancer drugs such as paclitaxel, etoposide, and cisplatin that rely on the p53 signaling pathway.]

p. 15, line 343.

[Collectively, these data support that the downregulation of AIMP2-DX2 in ML-1 cells can affect their response to anti-cancer drugs by modulating p53 signaling pathway.]

10. Page 23: "..database.21 (To) A.."- There is a typo.

[Response] We fixed the typo and proof-read the manuscript carefully to improve the writing.

REVIEWERS' COMMENTS:

Reviewer #1 (Remarks to the Author):

The authors have addressed the previous referees' comments in an adequate manner and the general quality of the manuscript has been substantially improved. I recommend publication of the present manuscript.

Reviewer #2 (Remarks to the Author):

The alterations of the text in response to the criticisms raised as well as the changes to the figures have improved the manuscript.

Response to Reviewers

We would like to thank the reviewers for the positive comments.

Reviewer #1:

The authors have addressed the previous refereesʹ comments in an adequate manner and the general quality of the manuscript has been substantially improved. I recommend publication of the present manuscript.

[Response] We thank the reviewer for the comment.

Reviewer #2:

The alterations of the text in response to the criticisms raised as well as the changes to the figures have improved the manuscript.

[Response] We thank the reviewer for the comments.
